# Recent Advancements in Stimuli Responsive Drug Delivery Platforms for Active and Passive Cancer Targeting

**DOI:** 10.3390/cancers13040670

**Published:** 2021-02-07

**Authors:** Muhammad Abdur Rahim, Nasrullah Jan, Safiullah Khan, Hassan Shah, Asadullah Madni, Arshad Khan, Abdul Jabar, Shahzeb Khan, Abdelbary Elhissi, Zahid Hussain, Heather C Aziz, Muhammad Sohail, Mirazam Khan, Hnin Ei Thu

**Affiliations:** 1 Department of Pharmaceutics, Faculty of Pharmacy, The Islamia University of Bahawalpur, Bahawalpur 63100, Punjab, Pakistan; muhammadabdurrahim88@gmail.com (M.A.R.); nasrullahjan14@gmail.com (N.J.); safiullahkhan856@gmail.com (S.K.); hasanshah342@gmail.com (H.S.); arshadpharma77@gmail.com (A.K.); 2 College of Pharmacy, University of Sargodha, Sargodha 40100, Punjab, Pakistan; jbkhan522@gmail.com; 3 Department of Pharmacy, University of Malakand, Chakdara, Dir Lower 18800, Khyber Pakhtunkhwa, Pakistan; dr.mirazam@uom.edu.pk; 4 Discipline of Pharmaceutical Sciences, School of Health Sciences, University of KwaZulu-Natal, Private Bag X54001, Westville 3631, Durban 4000, South Africa; 5 Division of Molecular Pharmaceutics and Drug Delivery, College of Pharmacy, The University of Texas at Austin, Austin, TX 78712, USA; 6 College of Pharmacy, QU Health and Office of VP for Research and Graduate Studies, Qatar University, P.O. Box 2713, Doha, Qatar; aelhissi@qu.edu.qa; 7 Department of Pharmaceutics & Pharmaceutical Technology, College of Pharmacy, University of Sharjah, P.O. Box 27272, Sharjah, United Arab Emirates; zhussain@sharjah.ac.ae; 8 Research Institute for Medical and Health Sciences (SIMHR), University of Sharjah, P.O. Box 27272, Sharjah, United Arab Emirates; 9 Division of Pharmacology and Toxicology, College of Pharmacy, The University of Texas at Austin, Austin, TX 78712, USA; heather.radford@utexas.edu; 10 Department of Pharmacy, COMSATS University Abbottabad Campus, Abbottabad 45550, Khyber Pakhtunkhwa, Pakistan; Msmarwat@cuiatd.edu.pk; 11 Research and Innovation Department, Lincolon University College, Petaling Jaya 47301, Selangor, Malaysia; eithuu287@gmail.com; 12 Innoscience Research Institute, Skypark, Subang Jaya 47650, Selangor, Malaysia

**Keywords:** tumor, chemotherapy, stimuli-responsive drug delivery systems, prodrugs

## Abstract

**Simple Summary:**

Cancer is one of the leading causes of death globally. Several studies, efforts and treatment strategies have been put forth for the treatment of different types of cancers. Several chemotherapeutic agents have been discovered and utilized for the treatment of various types of cancers and tumors, which play an important role in improving the quality of life of patients. The key problems associated with the abovementioned chemotherapeutic agents are the limited target ability and non-selective toxicity. The current review focuses on the achievement of improved targeting of anticancer agents at the tumor microenvironment without affecting normal tissues. The fulfilment of the mentioned objectives by stimuli-responsive drug delivery systems, as physical stimuli-responsive drug delivery systems and chemical stimuli-responsive drug delivery systems through active and passive targeting have extensively been discussed in the current review. The current review will help the wide community of researchers conducting research in targeted drug delivery systems and anticancer treatment strategies.

**Abstract:**

The tumor-specific targeting of chemotherapeutic agents for specific necrosis of cancer cells without affecting the normal cells poses a great challenge for researchers and scientists. Though extensive research has been carried out to investigate chemotherapy-based targeted drug delivery, the identification of the most promising strategy capable of bypassing non-specific cytotoxicity is still a major concern. Recent advancements in the arena of onco-targeted therapies have enabled safe and effective tumor-specific localization through stimuli-responsive drug delivery systems. Owing to their promising characteristic features, stimuli-responsive drug delivery platforms have revolutionized the chemotherapy-based treatments with added benefits of enhanced bioavailability and selective cytotoxicity of cancer cells compared to the conventional modalities. The insensitivity of stimuli-responsive drug delivery platforms when exposed to normal cells prevents the release of cytotoxic drugs into the normal cells and therefore alleviates the off-target events associated with chemotherapy. Contrastingly, they showed amplified sensitivity and triggered release of chemotherapeutic payload when internalized into the tumor microenvironment causing maximum cytotoxic responses and the induction of cancer cell necrosis. This review focuses on the physical stimuli-responsive drug delivery systems and chemical stimuli-responsive drug delivery systems for triggered cancer chemotherapy through active and/or passive targeting. Moreover, the review also provided a brief insight into the molecular dynamic simulations associated with stimuli-based tumor targeting.

## 1. Introduction

Cancer remains one of the leading causes of death globally. Each year more than eight million people die of cancer worldwide [1]. Despite the significant advancements in the fundamental understanding of cancer biology in the past several decades, the overall mortality of cancer is still high. A major reason for this is our incapability to deliver active pharmaceutical agents selectively to the disease sites without harming healthy tissue. Current treatment strategies for most cancers involve a combination of chemotherapy, radiation therapy and surgical resection. These treatment strategies are non-selective, which lead to significant morbidity and mortality. The primary concern with utilizing the chemotherapeutic agents is their inability to differentiate between healthy and cancer tissue. This is particularly harmful to rapidly growing cells in the body such as hair and soft tissues. The most cytotoxic agents are certainly the most effective but often result in severer adverse effects [2,3,4].

To get optimum effects, suitable drug dosage delivery and the duration of therapy in conjunction with a highly specified drug delivery system is required to be able to release the chemotherapeutic agents in a controlled manner. The major dispute of such a drug delivery system is to deliver the safe and optimum quantities of the drug compared to currently used treatment approaches [5]. Tumor targeting has emerged as an attractive strategy to allow access to tumors and avoid penetration into normal tissue interstitium. Tumor targeting is classified into passive and active targeting; however, the active targeting process occurs only after passive accumulation in tumors [6]. Passive targeting involves enhanced permeability and retention effect (EPR) due to rapid formation of hyper-permeable complex tumor vasculature characterized by impaired lymphatic drainage of diseased tissue (tumor), resulting in the extravasation of ≥100 nm nanoparticles into the tumor microenvironment and preventing their clearance. In contrast, the active targeting strategy is based on the composition decoration of the surface of drug carriers with tumor-specific ligands such as aptamers, antibodies and receptors overexpressed by the tumor cell [7].

**Figure 1:**
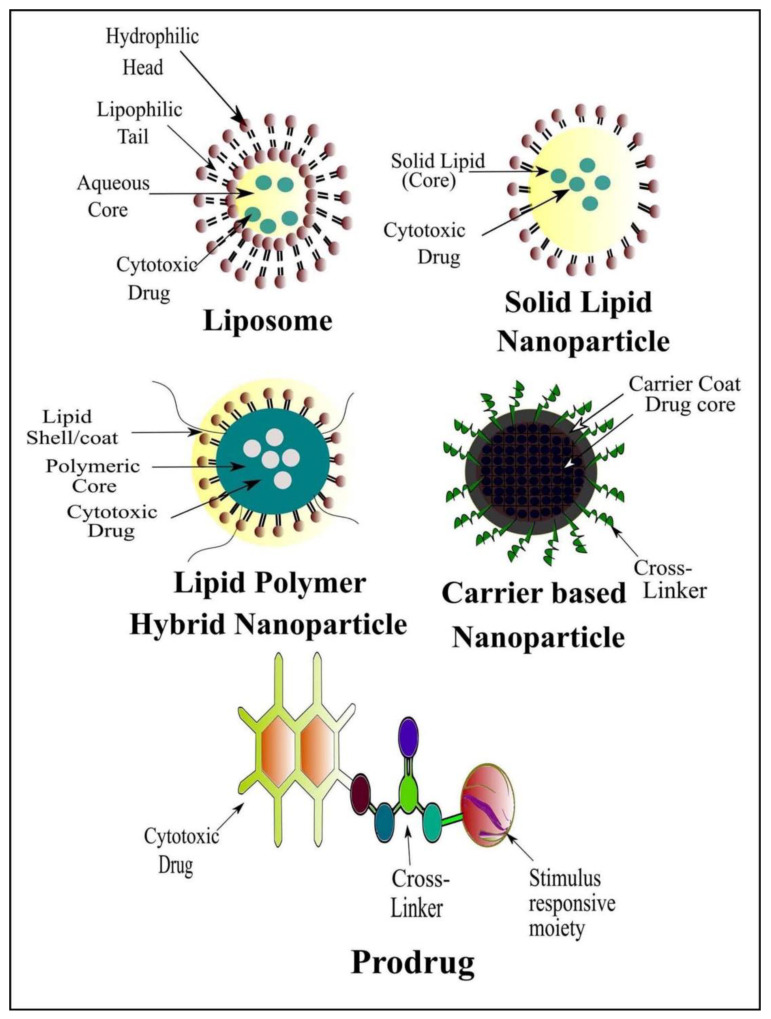
Renowned Stimuli-Responsive Drug Delivery Systems for Cancer Chemotherapy.

**Figure 2:**
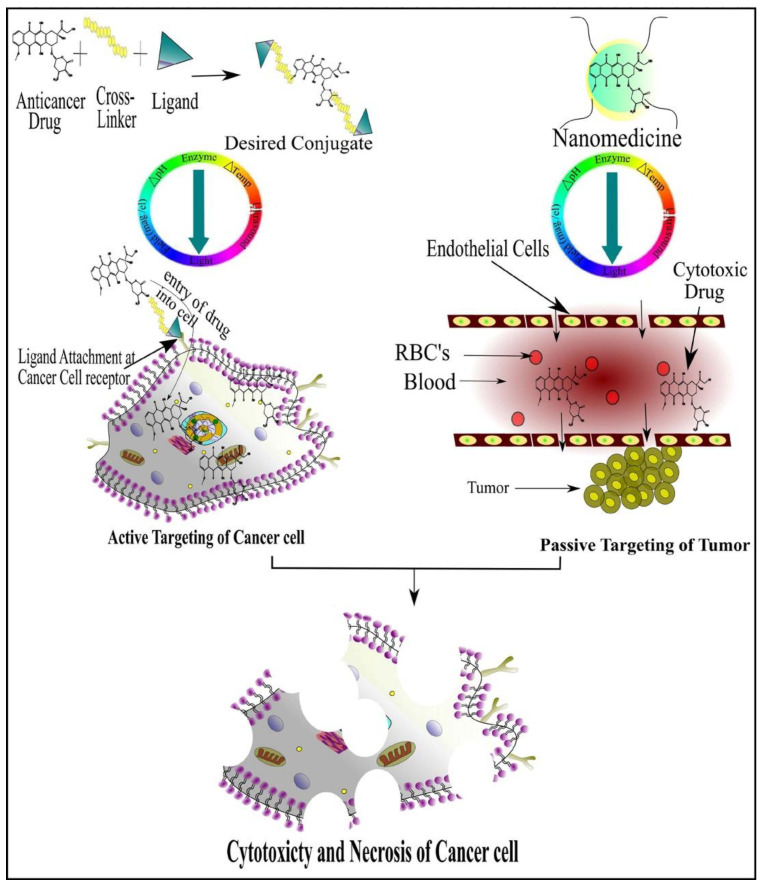
Cancer Cell-Specific Cytotoxicity by Active and Passive Targeting of Chemotherapeutic Agent(s).

Currently, great attention is being given from researchers to nanoscale-based drug delivery systems for the treatment of cancerous cells, such as tunable prodrugs [8], polymeric micelles [9], inorganic nanoparticles [10], nanotubes [11], nanorods [12], dendrimers [13], lipid-based drug delivery systems [14] and carrier-based drug delivery systems [15]. Among the aforementioned drug delivery strategies, stimuli-responsive lipid-based drug delivery systems, nanocarriers and prodrugs as displayed in Figure 1, have attained the greatest attention. Additionally, these drug delivery systems have the imperative attributes which include enhanced selectivity, biocompatibility, cancer microenvironment-based sensitivity and clinical acceptance with added benefits of easy scale up and multiple options regarding the choice and selection of formulation components. Drug carriers/lipids/prodrugs mostly exploit both passive and active strategies of tumor targeting to increase drug bioavailability. Drug delivery systems through stimuli-responsive carriers, lipids and/or prodrugs in the tumor milieu can lead to accelerated/triggered drug release at the target site, improved cellular binding and internalization, or more effective drug perfusion throughout the tumor volume [16]. The modern era has seen the development of multiple stimuli-responsive drug delivery systems that can achieve a several-fold enhancement of tumor necrosis. In addition, biocompatible excipients-based drug delivery systems are utilized for tumor-specific localization, stealth effect and easy clearance from the body. Moreover, advanced drug delivery systems could efficiently facilitate chemotherapy, gene therapy or both for theranostic applications of anti-neoplastics through passive targeting, active targeting, or combined active and passive targeting [17]. This review focuses on the stimuli-responsive drug delivery systems as physical stimuli-responsive drug delivery systems and chemical stimuli-responsive drug delivery systems for triggered cancer chemotherapy through active and passive targeting as presented in Figure 2. Moreover, this review also provides a brief insight into the molecular dynamic simulations associated with stimuli-based tumor targeting.

## 2. Stimuli-Responsive Drug Delivery Systems

Stimuli-based drug delivery systems have constituted a new platform in the understanding of diseases at the molecular level. For this purpose, nanotechnology-based drug delivery systems offer tremendous and promising advantages in the prevention, diagnosis and therapy of many diseases. The stimuli-based drug delivery system includes the phenomenon that influences an activity at a particular site or target tissue to bring about useful activities for the drug release via various mechanisms and is known as “stimuli responsive materials” or “environmentally-responsive materials” [18,19]. Stimuli-responsive materials are those that undergo a physical or chemical change in response to an external stimulus. These materials exhibit the environment responsive behavior phenomenon and respond to the external stimuli due to biomimetic nature. The stimulus-based drug delivery system is of great importance in the field of nanomedicines and nanotechnology due to controlled and targeted release at the site of action [20]. Stimuli-responsive systems respond to specific triggers to release their cargo at the desired site, hence they can enhance drug efficacy and overcome the adverse effects related to oral or parenteral drug delivery. Drug delivery systems with the ability to respond to temperature, pH change, enzymes, light, magnetic field, ultrasound or electrical stimuli have been heavily investigated over the past few decades [21]. Many intelligent designs of these delivery systems are based on polymers [22], hydrogels [23] and nanoparticles [24]. The stimuli-responsive drug delivery systems may be classified as physical and chemical stimuli-responsive drug delivery systems which are summarized in Table 1. 

### 2.1. Physical Stimuli-Responsive Drug Delivery Systems

#### 2.1.1. Thermoresponsive Drug Delivery Systems

Among different stimuli-responsive systems, temperature-responsive systems are the most investigated especially in the field of oncology [74]. In such a system, drug release is governed by variation in temperature of the tumor environment. Within normal body temperature (~37 °C), the thermoresponsive carriers retain the drug load. However, the drug is released at the local temperature of the tumor environment (~40–42 °C) [75]. A thermoresponsive drug delivery system is considered as an adjunct to hyperthermia therapy. In hyperthermia therapy, the body tissue is exposed to high temperatures via microwave, ultrasound or radiofrequency, which can kill or make the cancer cells more susceptible to specialized effects of radiation or chemotherapeutics agents. Along with the cancer cells, the hyperthermia therapies (microwave, ultrasound or radiofrequency) have toxic effects on the normal cells as well. Likewise, the chemotherapy also has toxic effects on normal cells as well as cancer cells. So, collectively, the combinations of chemotherapy and hyperthermia as a combinatorial strategy have toxic effects on the normal cells as well as cancer cells [76]. Therefore, thermoresponsive drug delivery systems that utilize the tumor microenvironment-based temperature, i.e., mild hyperthermia (~40 °C) which is not toxic for normal cells and causes the tumor-specific drug release, are proposed. Thermoresponsive systems are generally nanoparticles, liposomes or polymeric micelles that release the drug in appreciable rates only at targets having higher temperatures than normal cells of the body (e.g., cancer tissue) [74]. Thus, thermoresponsive liposomes, for example, function by minimizing the metabolism, uptake and clearance of drugs and concentrate the drug to the vasculature of the heated tumor. The released drug diffuses into the tumor, enhancing the drug concentration and the penetration at the tumor site. This approach does not depend upon passive targeting of the tumor. For abrupt release of the entrapped drug within the tumor, the thermoresponsive liposomes are administered during the mild hyperthermia treatment [75]. In thermoresponsive liposomes, lipids with suitable gel to liquid phase transition temperatures such as dipalmitoyl phosphatidylcholine or lysolipids are usually used [77]. Examples of such types of liposomes include doxorubicin-loaded thermoresponsive lysolipids-based liposomes (ThermoDox^®^, Doxil^®^, Myocet^®^) that have shown improved efficacy for cancer targeted delivery [78]. Thermoresponsive polymers exhibit lower critical solution temperature (LCST) and in response to LCST, the drastic change in aqueous solubility of polymers occurs. Below LCST, these polymers dissolve in water, whereas, above LCST, they are insoluble in water [79], get precipitated and disrupted the drug delivery system to release the drug [77]. Polymers such as poly (*N*-iso-propylacrylamide) (PNIPAAm) and poly(*N*-alkylacrylamide) compounds have been investigated as temperature-responsive polymeric micelles [80]. Block polymers can be synthesized by different polymerization techniques that consist of at least one thermoresponsive block with the ability to self-assemble in water to form thermoresponsive micelles. A copolymer can be synthesized by attaching a permanently hydrophilic block with a thermoresponsive one, which is hydrophilic below the LCST and micelles are formed once the transition in the phase of the thermoresponsive block occurs [81].

#### 2.1.2. Magnetic/Electric Field-Responsive Drug Delivery Systems

Magnetically responsive drug delivery (MRDD) involves entrapment of a drug in magnetic nanoparticles (MNPs); then, the drug/carrier complex is injected into the subject via intravenous (i.v.) or intra-arterial (i.a.) injection and finally, the complex is guided and concentrated at the desired locations by applying a high magnitude magnetic field externally. Once the carrier is concentrated at the desired site in vivo, the drug is released from the magnetic carrier, either via changes in physiological conditions, such as temperature, pH, and osmolality or through enzyme activity. This results in the limited systemic concentration of drug and increased localization at the tumor site [82]. Generally, a magnetic-responsive system consists of a core-shell system, in which the core contains magnetite (Fe_3_O_4_) or maghemite (Fe_2_O_3_), while the shell is composed of polymer, lipids, mesoporous silica or squalonyl-gemicitabine. A range of the investigated magnetic-responsive nanomaterials are superparamagnetic iron oxide nanoparticles (SPIONs) because of their ability to easily guide to the target site without retaining any residual magnetism, which at nanometer scale is attributed to quantum effects [83]. The multi-functional properties of magnetic nanoparticles can be achieved by either functionalizing the surface of nanoparticles with an organic shell (functionalized magnetic nanoparticles) or a system composed of thermoresponsive polymers loaded with the drug with implanted magnetic nanoparticles within their matrix (thermoresponsive magnetic nanocomposites) [84]. Magneto-liposomes, a combination of magnetic nanoparticles with liposomes are also introduced to control the liposome-based cargo release under magnetic stimuli [85].

The ideal magnetic-responsive drug delivery systems should ideally have the following properties [86]:

- Constituted particles at the nano-size range to allow perfusion at the capillary level;
- They should have adequate magnetic responsiveness;
- They should possess the ability to carry a wide variety of active therapeutic agents;
- They can be designed to function as controlled or targeted drug delivery systems;
- They have high biocompatibility and biodegradability and minimal antigenicity and toxicity.

The effectiveness of magnetic-responsive therapy is dependent on several physical and physiological parameters. The physical parameters include the strength and gradient of the magnetic field as well as volumetric and magnetic properties of the particles. Whereas the physiological parameters include a distance of the targeted tissue from the magnetic field, tumor volume, and source, strength and reversibility of the drug/carrier binding. As the route of administration of the carriers are normally intravenous or intra-arterial, hydrodynamic parameters such as rate of blood flow, concentration of the ferrofluid, circulation time and infusion route will also play a major role in the effectiveness of the magnetic therapy [87].

An electrical signal is considered as an actively controlled stimulus because it can easily be transported and does not require specialized equipment. The control of an electrical signal is easily done on demand and long cycles are possible. When a sensor or microchip system is combined with an electric signal, feedback and remote control from outside of the body can also be achieved [88]. An electric field responsive system consists of a pair of electrodes across the rate limiting membrane and polymer reservoir device. By changing the magnitude of the electric field between the electrodes, the drug release may be modulated and controlled in a predictable manner [89]. Nanoparticles based on conductive polymer (polypyrrole) showed a modified release of drug as a result of a combined effect of electrochemical oxidation-reduction and electric-field-responsive movement of charged molecules [90]. Electrically erodible polymers have been studied to control the release of insulin from mixtures of poly(ethyloxazoline) and either poly(acrylic acid) or poly(metha-crylic acid). A solid polymer complex is formed due to hydrogen bonding between both polymers. On the application of an electrical field, the changes of local pH at the cathode occur, causing hydrogen bonding in areas of the polymer adjacent to the cathode to begin to break down and consequently they begin to dissolve, resulting in a burst release of insulin. Removal of the electrical field halts the dissolution of the polymer and the release rate of insulin returns to the previously observed rate [91]. Electroporation is an efficient pathway for electro-responsive drug delivery which involves the application of a high voltage across the membrane to cause the formation of pores in cell membranes, thus, increasing their permeability to drugs [74]. This technique has been applied in gene therapy against cancer by using PEG-coated silica nanoparticles with opposite polarities [92]. Similarly, iontophoresis, which uses an electric field to increase the topical delivery of charged compounds is a particularly versatile approach in electro-responsive drug delivery [93]. Recently, this technique has been applied by Patil et al., 2020, for transdermal delivery of insulin using poloxomer gel [94]. It has become evident from this study that the potential drug candidates can be effectively delivered through a transdermal route using this technology.

#### 2.1.3. Ultrasound-Responsive Drug Delivery Systems (URDDS)

Ultrasound is used in different medical applications, such as imaging, kidney stone disruption, blood circulation analysis, lipectomy, tumor, leiomyoma and dentistry. Previously, low frequency ultrasound (LFUS) was mainly used to reduce the size of micro to nanoscale vesicles. Nowadays, ultrasound is used to induce targeted and controlled drug release [95]. Several physical effects are produced when an ultrasound wave propagates through tissue in the body, which can be used as triggers for ultrasound-responsive drug release. These physical effects include, acoustic fluid streaming, cavitation, simple pressure variation and local hyperthermia [96,97]. Cavitation is the most significant effect of ultrasound. In this effect, a large number of microbubbles are formed which grow and collapse during very short periods of time, when an ultrasonic wave transmits through a liquid medium. In acoustic cavitation, the permeability of a cell membrane can temporarily be changed to enhance the drug uptake by a process known as sonoporation [98,99]. In targeted therapy, URDDS has become an important research area. A wide range of drug delivery systems can be used to design URDDS including liposomes, emulsions, nanodroplets, nanobubbles, microbubbles, and micelles. Drugs that can be loaded into URDDS include biomacromolecules, small molecules and inorganic substances. URDDS are applied in different clinical conditions such as in the triggering of an immune response, treatment of Huntington’s disease, transdermal drug delivery, cartilage tissue engineering, treatment of ischemic myocardium, anticancer therapy, thrombolysis and disruption of the blood–brain barrier [100]. In cancer therapy, ultrasound plays a significant role due to its ability to be easily applied to a thermoresponsive system. Ultrasound produces hyperthermia via energy vibration through acoustic cavitation. These acoustic waves are applied as a release mechanism through cavitation which enhances the accumulation of anticancer drugs at the tumor site [101]. Recently, Xi et al., 2020, reported polymer-modified thermoresponsive liposomes (TSL) from utilizing a lipophilic anticancer chemotherapeutic agent for the treatment of cancer from TSLs [102]. This study reflects that this technology could be exploited for delivery of the challenging anticancer drugs using liposomal preparations.

#### 2.1.4. Light-Responsive Drug Delivery Systems (LRDDS)

Among different stimuli used for controlled drug release, light is considered to be a fascinating external stimulus because, with an on/off switching pulsatile behavior, drug release can be remotely triggered with high spatial and temporal precision [103]. LRDDS are mostly applied in photodynamic therapy (PDT), a combined therapy in which in the presence of tissue oxygen, light and photoactivatable photosensitizer are utilized. In PDT, the photosensitizer is injected intravenously and light-mediated activation converts the tissue oxygen to radical oxygen species (ROS). These ROS cause cellular necrosis, making PDT an ideal candidate for cancer therapy [104]. In PDT, photoreactions are induced by visible light or UV. UV or visible light is limited to topical treatments as applied to the skin or the mucosa. For deeper light penetration, i.e., more than a few millimeters, near-infrared (NIR) light within the range of wavelengths from 650 to 900 nm are mostly used in biomedical application [105]. Recently Xu et al., 2019, reported the NIR-based photosensitizers for photodynamic therapy [106].

#### 2.1.5. Stimuli-Responsive Lipids

**Figure 3:**
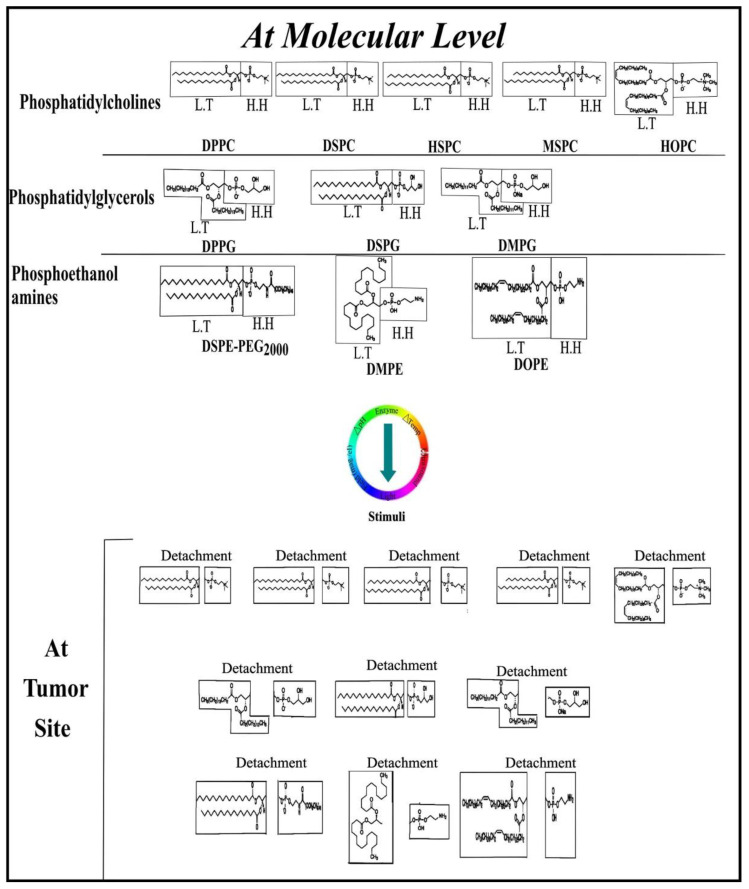
Molecular Transitions in Stimuli-Responsive Lipid-based Drug Delivery Systems used for Cancer Chemotherapy (“L.T” represents Lipophilic Tail, “H.H” represents Hydrophilic Head, “DPPC” represents 1,2-dipalmitoyl-sn-glycero-3-phosphocholine, “DSPC” represents 1,2-distearoyl-sn-glycero-3-phosphocholine, “HSPC” represents hydrogenated soy phosphatidylcholine, “MSPC” represents 1-myristoyl-2-stearoyl-sn-glycero-3-phosphocholine, “HOPC” represents 1-hexadecenyl-2-oleoyl-sn-glycero-3-phosphocholine, “DPPG” represents 1,2-dipalmitoyl-sn-glycero-3-phosphatidylglycerol, “DSPG” represents 1,2-distearoyl-sn-glycero-3-phosphatidylglycerol, “DMPG” represents 1,2-dimyristoyl-sn-glycero-3-phosphatidylglycerol, “DSPE-PEG_2000_” represents 1,2-distearoyl-sn-glycero-3-phosphoethanolamine-N-[amino (polyethylene glycol)-2000], “DMPE” represents 1,2-dimyristoyl-sn-glycero-3-phosphoethanolamine, “DOPE” represents 1,2-dioleoyl-sn-glycero-3-phosphoethanolamine).

**Figure 4:**
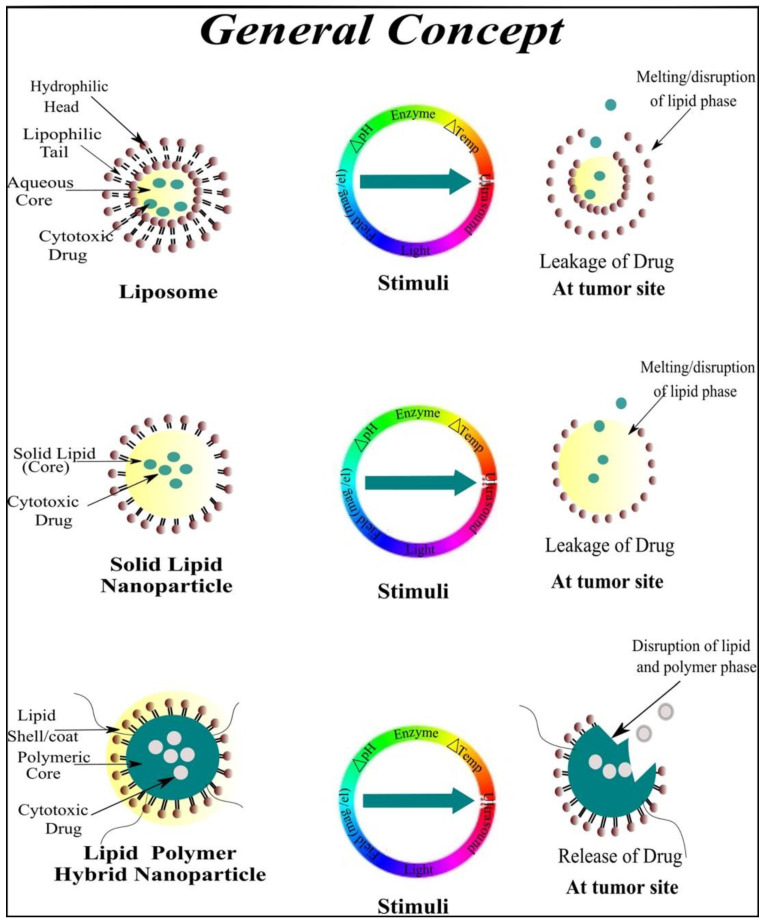
General Concept Regarding Stimuli-Responsive Lipid-Based Drug Delivery Systems Used for Cancer Chemotherapy.

The use of stimuli-responsive lipids within a lipid-based drug delivery systems is very imperative in anticancer therapy. Lipid-based drug delivery strategies include solid lipid nanoparticles (SLNs), nanostructured lipid carriers (NLC), lipid polymer hybrid nanoparticles (LPHNPs), liposomes, ethosomes and self nano/micro emulsifying drug delivery systems SNEDDS/SMEDDS and are entirely dependent on the use of biocompatible lipids. In addition, the undesirable side effects based on the non-specific distribution and targeting of chemotherapeutic agents to both normal and cancer cells also promote the use of biocompatible excipients (lipids) to achieve stealth effects and tumor-specific localization. The conventional chemotherapy causes a lack of tumor selective drug release, causing dose-limiting adverse effects and low bioavailability at the cancer site. To overcome these problems, drugs encapsulated in biocompatible lipids through I.V administration should be established to enhance bioavailability and promote selective targeting of tumor cells [107]. Stimuli-responsive lipids represent a unique class of lipids or amphiphiles used to induce transitions between a dissolution of the assembly or supramolecular states by the application of internal or external stimulus, e.g., temperature-responsive lipids, light-responsive lipids, pH-responsive lipids, sound-responsive lipids, electric field-responsive lipids and magnetic field-responsive lipids as illustrated in Figure 3 and Figure 4 [108,109]. Some of the commonly used stimuli-responsive lipids are listed in Table 2.

##### Temperature-Responsive Lipids

Thermoresponsive (temperature-responsive) lipids are favorable cancer treatment strategies as they efficiently utilize the dual advantage of the natural origin-based lipids and hyperthermia to improve the therapeutic effects and reduce side effects. The temperature at which the sol–gel lipids transition from gel to the liquid crystalline state is considered the phase transition temperature (T_c_) of the lipids as illustrated in Table 3 [134]. Different factors affect the phase transition temperature (e.g., the polar head groups and the length of the hydrocarbon chains of the different lipids like phosphatidylcholines (PC) and phosphatidylglycerols (PG)). For example, the T_c_ of dipalmitoyl phosphatidylglycerol (DPPG) and dipalmitoyl phosphatidylcholine (DPPC) are approximately 41 °C, whereas the T_c_ of dipalmitoyl phosphatidylethanolamine DPPE is 63 °C due to the difference in the polarity and length of the hydrocarbon chains. Likewise, the T_c_ of DSPC is 55 °C and that of DOPC is −20 °C due to the aforementioned type of differences [135]. Smet et al., 2010, [110] prepared three different types of doxorubicin-loaded temperature-responsive liposomes using different molar ratios of lipids such that DPPC:MPPC:DPPE-PEG_2000_ = 50:25:15:3 as high temperature-responsive liposomes, DPPC:MPPC:DPPE-PEG_2000_ = 86:10:4 as mild temperature-responsive liposomes and HSPC:Chol:DPPE-PEG_2000_ = 75:50:3 as temperature-insensitive liposomes. The mild temperature-sensitive liposomes showed abrupt doxorubicin release at around 38 °C, high temperature-sensitive liposomes showed abrupt doxorubicin release at 40 °C and slight doxorubicin release was observed from temperature-insensitive liposomes during heating based on the in vitro passive targeting of tumors. Lars H. Lindner (2004) [136] reported temperature-responsive liposomes with prolonged circulation time. The author reported the fabrication of different types of temperature-responsive liposomes using different ratios of phospholipids DPPC/DSPC/DPPGOG with 10% DPPGOG, 20% DPPGOG, 30% DPPGOG and without DPPGOG. The drug released was observed between 40 and 43 °C using molar ratios of 0–30% which showed fast and efficient drug release under minor hyperthermia. David Needham (2000) [137] also prepared doxorubicin-based temperature-sensitive liposomes using different molar ratios of HSPC:cholesterol:DSPE-PEG_2000_ based on non-temperature-sensitive liposomes (NTSL), DPPC:HSPC:cholesterol:DSPE-PEG_2000_, high temperature-sensitive liposomes (TTSL) and DPPC:MPPC: DSPE-PEG-2000 for low temperature-responsive liposomes (LTSL) for passive targeting of cancer cells. The LTSL showed increased doxorubicin release at elevated temperature of 42 °C (temperature associated with cancer cells), and in contrast, the NTSL and TTSL did not release any drug upon heating at 42 °C. Camptosar (CPT-11) and Cetuximab (CET) co-loaded dual responsive thermal and magnetic liposomes have been recently reported with strong anticancer activity in brain tumors cells. The liposomes were coated with magnetic Fe_3_O_4_ nanoparticles which identify the over-expressed epidermal growth factor (EGFR) on cancer cells that release the encapsulated drug when the liposomal temperature elevates by exposure to a high-frequency magnetic field that triggers the drug release on the surface of brain tumor cells [138]. In addition, thermoresponsive lipid-based drug delivery system can further be improved by combing with various functionalized nanomaterials such as thermoresponsive polymers, carbon nanomaterials and inorganic nanoparticles. Decoration of liposome surface with poly N-isopropylacrylamide (PNIPAM) or its copolymer can produce better thermoresponsive properties by hydrophobic interaction with the lipid membrane. Moreover, this could be elevated on a large number of animals for the safety profile at the efficacious dose by intravenous administration.

##### Electric/Magnetic Field-Responsive Lipids

The electric field can be used in a variety of therapeutic strategies that triggers drug release from the delivery system at the target site. Anticancer drugs, when combined with electric pulses, constitute a field referred to as “electro-chemotherapy”. The application of the external low electric field releases the active ingredients gradually. The electric field can also act on the cellular membrane, and along with stimulus-responsive abrupt drug release and cytotoxicity, it also may help to restore tissue reliability [147]. The lipid-based system represents a class of material which provides controlled release of active ingredients. An electric field-responsive system in the form of nanoparticles can be implanted or injected, which releases a cargo in the target tissues for a prolonged time upon local electrical stimulation. These drug delivery systems are ruptured by the electrically driven forces including electrofusion, electroporation, dielectric photophoresis, and electroosmotic assembly, which bring modification in the charges and potential energy of the lipid membrane. The addition of Poly (hydroxyethyl acrylate-co-hexadecyl acrylate-co-carboxyethyl acrylate) (P(HEA-HDA-CEA)) to the vesicular bilayer can produce thermo-electro-responsiveness as dual signal-responsive liposomes for passive targeting of cancer [148]. Kim et al., 2018 [118], also reported the abovementioned behavior of self-assembled liposomes through the use of P(HEA-HAD-CEA) in conjunction with egg phosphatidylcholine. The authors reported that the inclusion of the hydrophobic monomer hexadecyl acrylate (HAD) caused the achievement of LCST copolymer in aqueous solution. The copolymerization of hydroxyethyl acrylate (HEA) with hydrophobic monomer can show LCST in an aqueous solution. The HAD-inserted liposomes may cause the stasis of HAD, and by increasing the temperature of the simulated medium above phase transition temperature, cleavage of the polymer chain can happen and subsequent triggered release from the liposomes may occur. Similarly, an aqueous phase ionizable carboxyethyl acrylate (CEA) was included followed by applying an electric field, causing high shear stress in the vesicular bilayer, and tumor apoptosis at pH of cancer cells. Lim et al., 2009 [148], synthesized rupture-able and content release facilitator (over coplanar microelectrode arrays) liposomes by using different ratios of phospholipid 1,2-dipleoyl-sn-glycero-3-phosphocholine and Fluorescein-5-isothiocyanate (FITC “Isomer I”) combined with the capability of AC electroosmosis or dielectrophoresis. The aforementioned design controls the contents of liposome by micropatterned electrodes to achieve electrically controlled localized drug release at a predetermined position. The liposome rupture depends on the varying electric field strengths with a sensitive range of vesicle strengths over a micropatterned electrode array. This leads to the release of the drug contents at the target site in a controlled manner.

Likewise, magnetic liposomes or nanoparticles can be used as potential drug delivery systems for target-specific (tumor) magnetic field-based drug release. Pradhan et al., 2010, [116] synthesized calcein-loaded thermoresponsive and magnetic field-responsive liposomes for cancer targeting. The liposome formulations were designed by using doxorubicin hydrochloride with various molar ratios of phospholipid blends such as [1,2-dipalmitoyl-sn-glycero-3-phosphocholine (DPPC), cholesterol, 1,2 distearoyl-sn-glycero-3-phosphoethanolamine-N-[methoxy(polyethylene glycol)_2000_] (DSPE-PEG_2000_) and 1,2 distearoyl-sn-glycero-3-phosphoethanolamine-N-[folate(polyethylene glycol)_2000_ (DSPE-PEG_2000_-FA). The surface of the developed liposome was decorated with folate ligand for active targeting. Thus, temperature- and magnetic field-responsive folate-conjugated liposomes of doxorubicin were successfully developed which showed temperature and magnetic field responsiveness with an added benefit of folate receptors overexpression and uptake in tumor cells, resulting in improved active targeting of cancer cells. Li-peng Tseng [149] prepared magnetic field-triggered liposomes using various ratios of DPPC and cholesterol and 5 (6) –CF carboxyl fluorescein by using thin film hydration method for passive cancer targeting. The effect of magnetic field on the release of encapsulated drug was observed which showed the deformation of liposomal membrane under the influence of different magnetic field strengths: 0 tesla, 0.1 T, 0.2 T and 0.4 T. The liposomes obtained showed decreased deformability as liposomal stability increased. Similarly, the extent of cancer cell-specific disruption and fusion of liposomes was increased as the strength of magnetic field was increased. Redolfi Riva (2020), has reported doxorubicin-loaded magnetic/lipidic nanocarrier liposomes for actively targeting liver malignancies. The incorporation of magnetic nanoparticles in the liposomes increases uptake at the tumor site upon application of an external magnetic field. HepG2 cell line study showed significant selectivity in drug release at the target site. For monitoring the drug release within the cancer cells, the confocal microscopy confirmed fast DOX release inside the cancer cell [150]. Moreover, electric/magnetic field-responsive lipid-based drug delivery systems can be employed for different therapeutic approaches potentially increasing therapeutic efficacy. However, there is still a great need for further studies on behalf of the application of anticancer agents. The incorporation of gold nanoparticles on the surface of thermoresponsive liposomes can produce heat under light radiation and can achieve light trigger drug release at the tumor site. The incorporation of carbon nanohorns into liposomes can respond to temperature, light and magnetic field. Electric field-responsive nanohybrid liposomes with nanotubes can also be designed and controlled by an electric field.

##### Sound-Responsive Lipids

The ultrasound-triggered formulations are advanced drug delivery systems that function by enhancing the drug release and improving localization at cancer tissue [151]. The ultrasound can also efficiently control the release of drugs from liposomes with or without hyperthermia assistance. The release of the cytotoxic drug in this strategy is related to the use of low frequency ultrasound sources and their application on liposome membranes [152]. The presence of unsaturated acyl chains phospholipids and PEG-Lipopolymers improves the susceptibility of vesicle bilayers to low frequency ultrasound. The ultrasound-responsive liposomes were also prepared by different lipids in combination with perfluorocarbon DPPC/cholesterol/DSPE-PEG_2000_ [153]. Some phosphatidylcholine-based lipids as well as other phospholipids used in ultrasound-triggered drug release from liposomes have been discussed here. Frenkel and coworkers demonstrated that high-frequency intensity-focused ultrasound is an advanced non-invasive technique for targeted treatment by focusing a high energy focal point as well as high-frequency ultrasonic beams within the body [95]. Matos et al., 2019 [153], prepared ultrasound-responsive liposomes using various lipid ratios of DPPC/cholesterol/DSPE-PEG_2000_ and studied its release by high-intensity focused ultrasound (HIFU). Ultrasound-responsive liposomes were fabricated by mixing nanodroplets of perfluorocarbon (PFC) with pegylated liposomes. Perfluorocarbon undergoes phase alteration from liquid to gas when triggered with high-intensity focused ultrasound thus rupturing the lipid bilayer of ultrasound-responsive liposomes. The HIFU showed enhanced triggered release of drugs from USL and showed the highest release for 1–2 min after HIFU exposure time. The non-ultrasound-responsive liposome (NUSL) also showed triggered release with HIFU but considerably lower than the observed USL. NUSL also showed triggered release with HIFU but considerably lower than the observed USL. GUO, D., et al. prepared sound-responsive SLN-loaded nanoliposomes loaded with 1-bromomheptadecafluorooctane, hematoporphyrinmonomethylether (HMME) and doxorubicin (DOX) and explore their capabilities in releasing drugs by inhibiting HepG2 cells proliferation and generating reactive oxygen species (ROS) under low-intensity focused ultrasound (LIFU) for passive targeting of cancer cells. SLNs were fabricated and their basic features were tested and encapsulated in liposomes to form sound-sensitive nanoliposomes. The nanoliposomes showed approximately 84% release of DOX under LIFU irradiation for 8 h. The drug release can be prompted by LIFU in the nanoliposomes and enhances the inhibition of HepG2 cells proliferation [154]. For targeting the specific cancer cells, Sun et al., 2020 [155], reported the work of mimicking the mesenchymal stem cell membrane (coating material) and liposomes (core material). The developed mesenchymal stem cell membrane functionalized liposomal formulation delivered oxygen-loaded perfluorocarbon and verteporfin (sonosensitizer) to the target site. The sonodynamic therapy is a non-invasive anti-tumor approach that eradicates tumor through reactive oxygen species depending upon the mechanism involving the simultaneous interaction of chemical sono-sensitizer, low-intensity ultrasound and molecular oxygen. The sound-responsive lipids could be used for the biomimetic delivery of various types of cell membrane such as erythrocytes, leukocytes, neutrophils, cancer cells, and platelets which will ultimately target the cancer site and protect from phagocytosis.

##### Light-Responsive Lipids

Among the different types of stimuli-responsive drug delivery systems, photodynamic therapy has assisted a favorable treatment protocol that involves the use of a light source coupled with a photosensitizing agent. Light-responsive liposomes have been explored since the 1980s. For this purpose, photosensitive lipids have been used to form liposomes that immediately lose their stability when hydrolysis of the lipids is triggered by irradiation. The photosensitive lipids along with the photo-triggered system have the property to generate nano delivery systems for sustained and localized drug release [156]. Pramanik, S.K., et al., 2017 [157], prepared photosensitive hybrid liposomes using 1,2-bis(10,12-tricosadiynoyl)-sn-glycero-3-phosphoethanolamine (DTPE) as light-responsive lipid and cross linking facilitator in conjunction with DPPC and DMPC for the resultant functionalized hybrid liposomes. The liposomes formed by the incorporation of suitable functional end groups with lipids for conjugation of biomolecules can lead to the formation of nanocarriers enabling active targeting of antineoplastic agents and subsequent target-specific release. A phototriggerable liposome containing DPPC:DC_89_PC as vehicles for delivery of Glucoronide was prepared by Yavlovich et al., 2010 [156] (as mentioned earlier), containing various ratios of DPPC:DC_89_PC and 4 mol% DSPE-PEG_2000_.The drug release from these formulations was observed by photopolymerization of DPPC:DC_89_PC as proved by alteration in chromogenic properties of DC_89_PC. The resulting liposomes showed that UV treatment of DPPC:DC_89_PC of various concentrations caused photopolymerization of DC_89_PC and release of calcein, thus minimizing the potential toxicity of normal cells and leading to more effective cancer chemotherapy through the EPR effect. Recently, Liu, Y., et al., 2019, synthesized photothermal/photo-sonodynamic-mediated folic acid lipid nanoparticles for synergistic therapy in ovarian cancer and subcutaneous xenograft models. Lipid mixture containing DPPC, DPPG, DSPE-PEG2000, DSPE-folate-PEG2000, and cholesterol were used in the fabrication of lipid nanoparticles. This lipid nanoplatform has excellent potential as a promising strategy for the integration of dual mode imaging with photothermal/photo-sonodynamic therapy for the treatment of ovarian cancer. Light-sensitive lipid-based drug delivery can be further improved by attaching modality-responsive nano carriers. Incorporation of gold nanoparticles or gold nanoshells can generate heat under light irradiation and can achieve light-triggered drug release and imaging modality. Moreover, polymer-modified light-sensitive liposomes can also be prepared for adjuvant properties, targeting properties and immunity-induced functions depending on the backbone structure in the light-responsive groups.

##### pH-Responsive Lipids

The pH of the pathological tissues such as inflammation, infection and cancer is considerably different from the pH of normal tissues. The pH at the tumor site is lower than that of normal tissues [158]. Different types of pH-responsive liposomes have been proposed constituting combinations of phospholipids or compounds containing an acidic group that act as a stabilizer at neutral pH. Simões et al., 2004 [159], used different types of lipids for pH-responsive liposomes such as DSPC, HSPC, DPPE with or without cholesterol and lipid conjugates such as phosphatidylethanolamine—poly(ethylene glycol) (PE–PEG) which decreases the leakage of encapsulated drugs in the extracellular milieu or during circulation. Moreover, it is based on the nonspecific interaction and showed efficient delivery of anticancer drugs and proteins at a specific pH. Likewise, Hong et al., 2002 [146], prepared a pH-responsive, serum stable long circulating liposome by using dioleoylphosphatidylethanolamine (DOPE) and oleic acid or DOPE and 1,2 dipalmitoylsuccinylglycerol (DOPE/DSPG). The serum stability of DOPE/oleic acid and (DOPE/DSPG) was enhanced by DSPE-PEG_n_. In the tumor microenvironment where the pH is lower than the normal healthy tissues, the DOPE/DSPG liposomes released the drugs more rapidly than liposomes with traditional compositions. Moreover, the use of DSPE-PEG_n_ significantly increased the circulation time of methotrexate. The use of DOPE/DSPG and DSPE-PEG_n_-based liposomes was plasma stable, pH-responsive and had long circulation time. Shi et al., 2002 [160], prepared pH-sensitive cationic or anionic liposomes incorporating folate molecules. The pH-responsive liposomes composed of dimethyldioctadecylammonium bromide (DDAB), egg phosphatidylcholine, cholesterol hemisuccinate and Tween 80 showed rapid drug release only at acidic pH. These liposomes showed improved pH sensitivity in the presence of serum compared to conventional pH-responsive liposomes. The incorporation of 0.1 mol % folate-polyethyleneglycol-phosphatidylethanolamine (f-PEG_n_-PE) facilitates FR-mediated endocytosis of liposomes. The FR-targeted pH-responsive liposomes showed increased cytosolic release of entrapped calcein and enhanced cytotoxicity compared to non-FR-targeted and conventional pH-responsive liposomes. These findings showed that FR-targeted pH-responsive liposomes are effective vehicles for intracellular gene and drug delivery through dual and passive mechanisms of drug targeting.

pH-responsive lipid-based systems can be engineered with both pH and light trigger release capabilities of the loaded anticancer drugs. This technology has become a very popular approach and recently one of the studies demonstrated that DOX/FOBD55 resulted in a significant anticancer effect using a mouse model compared to the control group [161]. Over the past several decades, pH-responsive drug delivery systems have contributed to the momentous progress regarding tumor targeting and cancer therapy, but still there is a need to develop the understanding of in vitro and in vivo acceptability and behavior, optimum composition and excipients and more importantly the in vivo clinical applications. Incorporation of additional organic/inorganic carriers and other novel nanostructured materials can increase the loading capacity and enable simultaneous bio imaging, multimodal therapy, surface modification, sensing and tissue engineering.

##### Enzyme-Responsive Lipids

The prime principle behind targeted drug delivery is to maximize therapeutic effects while minimizing adverse effects. To avoid possible toxicity, the delivery of therapeutic agents to the non-target sites should be reduced. The targeting of nanoparticles can be improved by grafting certain stimuli-responsive molecules onto the nanoparticle surfaces. The physical properties of nanoparticles can be personalized by a multidisciplinary task to precisely determine the choice of drug release at the specific site and the related biological interactions [162]. The development of bio-responsive nanomaterials to the bio-catalytic action of an enzyme is an emerging field in the nanotechnology-based drug delivery strategies. Enzymes are important to target therapeutics at specific sites as they play a pivotal role in cell regulation. The nanomaterials can be programmed in such a way that when an enzyme is found in higher concentrations at the target site or the magnitude of enzyme activity is increased at a specific tissue, the nano-carriers then release the entrapped drug. The enzyme-responsive nanomaterial has led to growing interest in biological applications such as therapeutics and diagnostics which may either be due to its predictable chemical composition as a biocatalyst or by modification in enzymatic reactions [163]. Compared to conventional drug delivery systems, enzyme-responsive nanoparticles are advantageous for drug delivery, with improved features. This may provide site-specific delivery through programming the nanoparticles that releases the drug by eliciting enzymatic decomposition of the nanocarrier. In chemotherapy, the toxicological problems associated to hazardous drugs can be reduced by a specific triggering of the drug release and cargo due to the biological recognition of the substrate by the enzyme. The different types of enzyme-responsive nanoparticles are liposomes, polymer-stabilized liposomes, hybrid nanoparticles, polymeric nanoparticles, gold nanoparticles and others [164]. Bruun et al., 2015 [131], prepared enzyme-responsive titratable cationic Lipid based Nanoparticles (LNPs) for delivery of siRNA across the blood brain barrier (BBB) for targeting glioma cells. Stable, safe and effective delivery of siRNA to the CNS is conditional upon the ability of the formulation to cross the BBB. The LNPs effectively encapsulate siRNA and the delivery is achieved through two phases; first by targeting the low-density lipoprotein receptor to BBB by conjugation of angiopep to the surface of LNP, and secondly, a negatively charged polyethyleneglycol cleavable lipopeptide is used to mask the positively charged LNPs which contain a class of enzymes mostly expressed in inflammatory BBB and in the tumor microenvironment (active targeting). The LNP thus favors the release of siRNA with high efficiency by changing from negative to the positive charge. Pourhassan et al., 2017 [132], prepared oxaliplatin (L-OHP), a platinum drug in phospholipase A_2_ (sPLA_2_)-sensitive liposomes for cancer therapy using negatively charged phospholipid desired to achieve enzyme sensitivity. The cell cytolysis is governed by the extent of lysolipids and the serum protein and the sensitivity of the formulation to the enzyme. In vivo efficiency investigated on nude mice after intravenous injections resulted in tumor suppression using liposomal L-OHP in comparison to free drugs that showed a weak response. This indicates that phospholipase A_2_ (sPLA_2_) triggered the release of the drug and caused cytotoxicity of cancer cells through both active and passive targeting. A recent study has been put forth by Kong et al., 2020, who synthesized vinorelbine- and dioscin-loaded liposomes for the treatment of non-small cell lung cancer (NSCLC). The liposomes were responsive to overexpressed MMP2 (matrix-metalloprotease-2) enzymes, resulting in enhanced tumor targeting, internalization and enhanced anti-tumor efficacy both in vitro and in vivo. The reported novel liposomes may provide a potential platform regarding the antitumor treatment strategy for NSCLC [165]. Enzyme-responsive lipid-based drug delivery is useful regarding anticancer therapy, but still, many challenges need to be addressed. Efficient delivery of siRNA can be made which can efficiently increase the uptake and gene knockdown by incorporating the MMP-cleavable lipopeptides in the lipid-based formulations. Moreover, an angiopep functionalization can access transport through BBB and could play an important role in treating glioma through enhanced EPR and active targeting. Some of the lipid based drug delivery systems and prodrugs remained successful in the achievement of FDA approval. The FDA approved Lipid based drug delivery systems and prodrugs have been illustrated in Table 4 below.

### 2.2. Chemical Stimuli-Responsive Drug Delivery Systems

#### 2.2.1. pH-Responsive Drug Delivery Systems

The pH value in different tissues and cellular compartments varies inside the body (Table 5). These variations of pH in different diseased conditions such as inflammation, infection, ischemia and cancer can be exploited for pH-responsive drug delivery systems [190,191]. In tumor tissue, within the interstitial matrix, the metabolic profile is different due to poor perfusion of oxygen, resulting in a high level of lactic acid and pH drop from 7.4 to 6 (slightly acidic). The changes in pH can be exploited for drug targeting in two ways: (1) by targeting the extracellular tissue, where pH ranges between 6.5 and 7.2 or (2) by targeting the lysosomes with pH range of 4.5–5.0. In lysosome targeting, hydrolytic enzymes such as cathepsin B may also be utilized to release the drug [192]. The pH-responsive systems must incorporate pH-tunable moieties. These moieties can utilize a variety of functional groups such as hydrazone, ortho ester, amine, acetal and vinyl ether, which function as pH sensors because their insolubility in water is changed by protonation and deprotonation [190,193]. Different delivery systems for achieving pH-responsive drug release have been reported such as liposomes [194], polymers [195], micelles [196] and dendrimers [197]. Hira et al., 2020, has recently developed doxorubicin-loaded pH-responsive nanomicelles for treatment of murine lymphoma. The drug was effectively released from the nanomicelles at low pH conditions, i.e., tumor microenvironment [198] as expressed in Table 2. In vivo results showed pH-responsive nanomicelles loaded with doxorubicin have a prolonged blood resident time owing to minimal drug leakages.

#### 2.2.2. Enzymes-Responsive Drug Delivery Systems

Enzymes are linked with different pathophysiological and physiological processes in the human body. In cancer, enzymes are mostly associated with tumor growth, progression, angiogenesis, extravasation, intravasation and metastasis. In different types of cancer, elevated levels of specific enzymes occur. This elevated expression of specific enzymes can be exploited to achieve enzyme-responsive drug release [200]. Due to the selective catalytic action of enzymes, enzyme-responsive systems undergo reversible macroscopic transitions. When enzyme-responsive materials (ERMs) are triggered through enzymes, then the changes in intra- and inter-molecular interaction occur with the subsequent macroscopic transition. Some of the reported macroscopic transitions in these materials include: sol to gel, gel to sol, suspension to gel transitions and disassembly of nanoparticles which are visualized through change in colors. An enzyme-responsive system consists of an enzyme-responsive component (such as a peptide, polynucleotide or lipid) and a component that controls and directs molecular interactions that cause macroscopic transition [201]. Matrix metalloproteinases (MMPs) are the most important extracellular enzymes associated with cancer growth and metastasis. In the tumor microenvironment, MMPs are considered as robust stimuli for enzyme-responsive drug delivery and tumor targeting. The MMP-responsive nanocarriers remained inactivated in normal tissues and blood due to low levels of MMPs and stability of nanoparticles and MMP substrates. While in the tumor, due to an upregulation of MMPs, the catalysis of MMP substrate occurs, resulting in drug release into the tumor microenvironment [202]. Hu et al., 2016 [47], developed MMP-responsive mesoporous silica nanoparticles for theranostic application. In this study, the surface of mesoporous silica nanoparticles was decorated with MMP-responsive peptide for diagnostic purposes as well as for tumor targeting. In tumor tissue with overexpressed MMP-2, tumor imaging and triggered drug release occurred due to the hydrolysis of the MMP-2-responsive peptide substrate, while in the absence of MMP-2, no fluorescence and drug release occurred. Besides MMPs, some other specific enzymes (cathepsin B, lipases, urease, oxidases and peroxidases) can be explored for controlled drug release applications [203].

#### 2.2.3. Stimuli-Responsive Prodrugs

**Figure 5:**
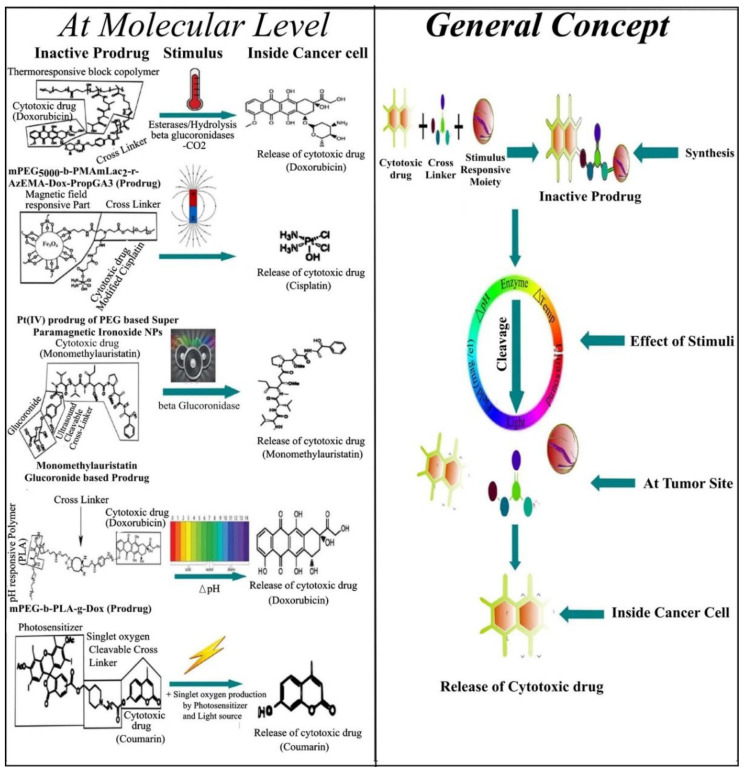
Stimuli-Responsive Prodrugs for Cancer Chemotherapy. “mPEG_5000_-b-(HPMAmLac2-r-AzEMA)-DOX-PropGA3” represents poly(ethylene glycol)_5000_-b-poly[N-(2-hydroxypropyl) methacrylamide-lactate]2-2-azidoethyl methacrylate-doxorubicin propargyl glucuronide A3 prodrug, “mPEG-b-PLA-g-DOX” represents mPolyethyleneglycol-b-norbornene functionalized polylactic acid-grafted doxorubicin.

The prodrug approach includes the inactive precursors of the active drug that can be activated/bioconverted to improve the pharmacokinetics of the drug. The improved pharmacokinetic properties include oral absorption and intestinal permeability. Prodrugs are selectively targeted to the tumor site either by passive targeting or active targeting. Some of the prodrugs remain successful in achieving FDA approval, as described in Table 4. The stimuli-responsive cross-linked prodrugs are another versatile approach for the cancer chemotherapy through active and passive targeting. Stimuli-responsive prodrugs represent a promising drug delivery approach and attract scientific attention regarding tumor targeting and in the management of a variety of malignancies. The aforementioned fact is associated with stimuli-responsive prodrugs-based drug delivery strategies with added benefits of enhanced sensitivity towards neoplasia, limited or no sensitivity towards hyperplasia and normal body cells, improved tumor-specific localization, enhanced tumor uptake and achievement of cancer cells apoptosis and necrosis through active and passive targeting or both. The stimulus sensitivity may be achieved through the cross linker, cross-linked agent(s) or through physical combination with the cytotoxic drug. Moreover, the single stimulus-responsive prodrugs, dual stimuli-responsive prodrugs and even triple stimuli-responsive prodrugs associated with the cancer cell microenvironment through active targeting, passive targeting or dual active and passive targeting for chemotherapy, gene therapy and theranostic applications could be achieved through a prodrug-based drug delivery strategy [204]. The detailed discussion about the stimuli-responsive prodrugs such as temperature-responsive prodrugs, light-responsive prodrugs, magnetic/electric field-responsive prodrugs, ultrasound-responsive prodrugs, pH-responsive prodrugs and enzyme-responsive prodrugs [205] have been discussed in this review and are illustrated in Figure 5 and Table 6.

##### Temperature-Responsive Prodrugs

Temperature-responsive prodrugs are prodrugs which release the drug at a specific temperature or temperature range and prevent the drug release at other temperatures. Talelli et al., 2011 [229], reported the development of thermoresponsive polymeric micelles coupled with a glucuronide-based prodrug for dual active and passive targeting. In brief, 2-Azidoethyl Methacrylate monomer was synthesized from 2-Azidoethanol (mixture of sodium azide and 2-bromoethanol in the mixture of organic solvent followed by removal of organic solvents). The subsequent conversion into 2-Azidoethyl Methacrylate monomer was achieved by esterification reaction of 2-Azidoethanol and methacroyloyl chloride. Moreover, the functionalization of copolymer (HPMAmLac_2_) followed by coupling with propargyl derivative of the glucuronide-based doxorubicin prodrug was done. The reaction was carried out using 2-azidoethanol as monomer and (mPEG_5000_)2-ABCPA as radical micro initiator. In short, the aforementioned prodrug was achieved through free radical polymerization and click chemistry methods. The dual targeting has been reported through the prodrug. The polymeric micelles caused the tumor-specific hyperthermia-based extravasation by EPR effect. On the contrary, the prodrug caused the overexpression of human beta-glucoronidase (enzyme at tumor site) followed by intracellular entry of doxorubicin and subsequent cell death [206]. Han et al., 2014 [209], reported the development of dual light and temperature-responsive cypate containing reduction-responsive platinum IV [Pt(IV)]-based polymeric prodrug micelles to overcome the resistance of cancer (A549R) cells against chemotherapeutic agents. Initially, the alkynyl functionalization of Pt(IV) and cypate (near infra-red fluorescent dye) was achieved by EDC.HCl (N-(3-dimethylaminopropyl)-N-ethylcarbodiimide hydrochloride) coupling reaction. In addition, the synthesis of macroRAFT chain transfer agent [P(MEO_2_MA-co-MASI)] was achieved by using P[MEO_2_MA] (poly[(2-(2-methoxyethoxy)ethyl methacrylate) and MASI (N-methacryloxy succinimide) using CTP (4-cyanopentanoic acid dithiobenzoate) as chain transfer agent. After that, synthesis of block copolymer P(MEOMA-co-MASI)-b-PHPMA was established by RAFT polymerization of HPMA (N-(2-hydroxypropyl)methacrylamide) with [P(MEO_2_MA-co-MASI)]. The aforementioned reaction continued with conversion of block copolymer into P(MEO_2_MA-co-MASI-coAzPMA)-b-PHPMA through amidation reaction and azide functionalization (3-azidopropylamine). In the final step, the alkynyl functionalized Pt(IV) and cypate were conjugated with P(MEO_2_MA-co-MASI-coAzPMA)-b-PHPMA through click chemistry method and P(Pt-Cyp-MEO_2_MA-co-MASI)-b-PHPMA micelles achieved. It has been reported that the synergistic effects of Pt(IV) prodrug and cypate in the conjugated form with the reduction sensitive polymeric micelles resulted in triple (light, temperature, reduction) responsive prodrug-micelles. Moreover, the achieved prodrugs can enhance stealth behavior, sensitization, uptake and apoptosis of cancer cells against anticancer agents through passive targeting. Yu et al., 2015 [52], reported the development of pluronic 123-conjugated doxorubicin prodrug micelles (P-DOX) and cypate-conjugated poly(ethylene glycol)-block-poly(diisopropanolamino ethyl methacrylate), i.e., (P-cypate) for passive targeting. In short, initially P-DOX was achieved by glycine-phenylalanine-leucine-glycine conjugation reaction, whereas, P-cypate diblock copolymer through atom transfer radical polymerization and coupling reaction. The aforementioned types of micelles were combined through mixing of developed micelles followed by solvent evaporation technique. It has been reported that cypate alone could also provide dual light and hyperthermia-assisted drug release but synergistic photothermal and pH-responsive-stimuli-based drug release was observed when converted to (P-cypate/P-DOX) prodrug, respectively. Li et al., 2016 [207], reported the successful achievement of IR-780-loaded polymeric prodrug micelles for tumor-specific passive targeting of doxorubicin through EPR to achieve successful apoptosis and necrosis of cancer cells. The polymeric prodrug micelles of IR-780-loaded poly(methacryloyloxyethylphosphorylcholine)-b-poly(2-methoxyethylmethacrylate)-hydrazide-doxorubicin) were synthesized by initial synthesis of poly(*n*-butylmethacrylate)-*b*-poly(methacryloyloxyethyl phosphorylcholine) block copolymer by atom transfer radical polymerization. The final product was achieved by dissolving IR-780 and poly(methacryloyloxyethyl phosphorylcholine)-b-poly(2-methoxyethyl methacrylate)-hydrazide-doxorubicin) in organic solvents followed by removal of organic solvents and subsequent dialysis against distilled water. Authors reported that the obtained micelles showed enhanced photothermal ablation assisted stimuli-based uptake and in vitro as well as in vivo apoptosis of cancer cells. Likewise, Zhang et al., 2016, reported the photothermal stimulus-based dual active and passive targeting of doxorubicin through the use of folic acid and NIR dye in the conjugated form with prodrug. Initially, the macroRAFT agent (P[monomethoxy oligo(ethylene glycol) methacrylate)] was synthesized by RAFT block copolymerization of methacrylamide tertbutyl carbamate using monomethoxy oligo(ethylene glycol) methacrylate) as a monomer and the aforementioned macroRAFT agent to achieve P[methacrylamide tertbutyl hydrazide]-b-P[moligo(ethylene glycol) methacrylate)] diblock copolymer. The developed diblock copolymer was then conjugated with folic acid (PMABH-b-POEGMA-FA) followed by treatment with trifluoroacetic acid to achieve PNHNH_2_-b-POEGMA-FA, and finally conjugation with DOX.HCl to achieve the desired prodrug. The developed prodrug was then converted into prodrug nanoparticles such that the IR-825 was entrapped in the hydrophobic core of the prodrug nanoparticles (PDOX/IR-825). In another study, the authors reported the development of prodrug-based hollow mesoporous silica nanoparticles in which the doxorubicin prodrug acted as a coat of IR-825-entrapped hollow mesoporous silica nanoparticles. In all of these aforementioned studies, it has been reported that a minimal amount of drug release occurred under physiological conditions, while maximum in vitro drug release was achieved at the pH of cancer cells through laser irradiation of PDOX/IR-825 nanoparticles. Likewise, the study carried on by the same authors in 2018, revealed successful development of IR-825-loaded hyaluronic acid-modified polymeric prodrug nanoparticles for photo-thermal and reduction sensitivity-based enhanced apoptosis of the tumor site through a passive targeting mechanism [210,230].

A recent study has been carried out by Du et al., (2019) [231] and reported the achievement of a dual drug-paired polyprodrug nanoparticle (PDCN_25_-CDDP) for photo-thermal stimuli-based cancer therapy. The developed nanoparticles showed enhanced photo-thermal stimulus-based drug release at tumor conditions, while negligible drug release was observed at normal physiological conditions both in vitro and in vivo. All aforementioned photo-thermal stimuli-derived prodrugs associated with cancer therapy were based on the near infra-red light (NIR) sensitizer in conjunction with an anticancer drug, such that the laser irradiation of NIR provided light and high temperature as well. Although enormous work has been carried out in the field of thermoresponsive prodrugs for cancer therapy, applied research is still lacking in this field. So, in near future, the applied research and/or human volunteers-based clinical trials should be initiated for the verification associated with safety and efficacy of anticancer agent(s)-loaded thermoresponsive prodrugs in humans.

##### Magnetic/Electric Field-Responsive Prodrugs

Electric/magnetic field-responsive prodrugs are used for theranostic application against tumors through the use of magnetic/electric field contrast agents in combination with cytotoxic agents for cancer cell-specific targeting and apoptosis/necrosis. Although very few studies have been reported regarding field-responsive prodrugs for cancer therapy, this subject could be considered as a current gap in cancer research. Frullano et al., 2006 [211], reported the development of a prodrug containing doxorubicin in conjunction with Gadolinium(III) as a magnetic resonance imaging (MRI) contrast agent for theranostic application. It has been reported that MRI contrast agent was covalently attached to doxorubicin (at carbon 13, through the hydrazone group) and the desired conjugate was achieved through a series of reactions from tert-butyl-protected DO_3_A. In addition, the synthesized product released the drug in vitro at the pH of cancer by hydrolysis, while it remained stable at normal physiological pH. Hernández-Gil et al., 2015 [212], reported the development of Pt(IV) prodrugs in combination with magnetic field-responsive iron oxide nanoparticles. Briefly, the succinic acid derivative of Pt(IV) based prodrug was formed, followed by converting the prodrug into DSPE-PEG_2000_-Pt(IV) mixture through coupling reaction using DSPE-PEG_2000_-NH_2_ and freeze drying [209]. The freeze-dried DSPE–PEG_2000_–Pt(IV) complex was then converted into DSPE–PEG_2000_–Pt(IV) prodrug-based iron nanoparticles by dissolving in organic solvents followed by removal of organic solvents and subsequent stirring, ultracentrifugation and filtration, respectively. Finally, the prodrug-based nanoparticles were functionalized through a cytotoxic double stranded RNA analog (polyinosinic–polycytidylic acid). The authors reported that Pt(IV) prodrugs alone were non-cytotoxic, whereas, Pt(IV) prodrugs and double stranded RNA analog-based prodrugs nanoparticles were theranostic in nature as iron nanoparticles act as MRI contrast agents and RNA analogs were onco-cytotoxic. Zhu et al., 2016 [213], also reported the development of Pt(IV) prodrug-based iron nanoparticles with slight modification for the achievement of nanocomposites for theranostic application. The succinic acid derivative of Pt(IV) prodrug was modified by oxidizing cisplatin with hydrogen peroxide and using PEGylated iron nanoparticles. The modified Pt(IV)-based prodrug was then conjugated with PEGylated iron nanoparticles through EDC coupling chemistry and Pt(IV). The prodrug was coated over the PEGylated iron nanoparticles for the achievement of nanocomposites. Moreover, the author revealed the boosted cytotoxicity of cancer cells by glutathione through dual active and passive targeting and minimal cytotoxicity of normal cells. Lopez et al., 2003 [214], reported the development of 5-aminolaevulinic acid (ALA) in conjunction with an iontophoresis-based prodrug as electric field=responsive prodrug therapy for skin cancer. Three sets of experiments were done such that, initially the preferable electrode selection was determined by the application of ALA buffer solution on different electrodes followed by a selection of suitable amounts of electrode and drug and subsequent comparison with the drug. The author reported that skin was exposed to the optimized formulation with suitable iontophoretic flux, drug concentration and electrode (anode) and fourfold enhanced delivery of ALA was achieved compared to the drug.

Yang et al., 2019 [232], has recently exploited the magnetic field-responsive prodrugs for theranostic applications. The developed prodrug was containing a synthesized compound associated with enhanced MRI contrast property and Gadolinium as a diagnostic agent in conjugated form. Moreover, the aforementioned prodrug presented improved cancer cell uptake, enhanced cancer cell specific cytotoxicity and biotin onco-receptors overexpression for dual active and passive targeting of tumors. In the future, a variety of safe and biocompatible electric field- and/or magnetic field-responsive diagnostic agents and therapeutic agents-based prodrugs could be utilized for a diagnostic, antitumor and theranostic applications for the management of various types of cancers.

##### Ultrasound-Responsive Prodrugs

The ultrasound-responsive prodrugs are also used for theranostic application by utilizing ultrasound source, ultrasound contrast agents and anticancer agents for tumor-specific localization and apoptosis/necrosis. Aoi et al., 2008 [233], reported the ultrasound and ganciclovir-loaded nanobubble for cancer and gene therapy through herpes simplex thymidine kinase (HSV*tk*) gene transfer. Two ultrasound probes with an amplifier, hydrophone and oscillation detector were used during the study. It was observed from the in vivo study that herpes simplex thymidine kinase (HSV*tk*) gene in conjunction with ganciclovir, ultrasound and nanobubble showed fourfold enhanced cancer cell apoptosis and less cytotoxicity to normal cells. Bezagu et al., 2017 [234], reported the development of ultrasound-activated anticancer drug release to achieve theranostic use of antineoplastic agents. An ultrasound-assisted (transductor with detector) glucuronide-conjugated antimitotic agent monomethylauristatin E(CH_3_-Auristatin-E-glu) was selected as a prodrug for the study to achieve tumor-specific cytotoxicity, as mitotic agent alone causes the non-specific cytotoxicity. In extension, the beta-glucoronidase was also used in conjunction with ultrasound and CH_3_-Auristatin-E-glu perfluorocarbon (ultrasound-responsive active targeting capable) composites as a combinatorial strategy for enhanced in vitro cytotoxicity. Luo et al., 2017 [215], reported dual active and passive tumor-specific targeting of doxorubicin through pH and ultrasound-responsive folate-conjugated doxorubicin prodrug microbubble complex. The prodrug was achieved through initial conjugation of PEG and cyclic RGD peptide followed by conjugation with heparin, folic acid and biotinylated doxorubicin to achieve Heparin-FA-PEG-cRGD-DOX. The synthesized conjugate (prodrug) was then reacted with biotinylated microbubble and finally, doxorubicin microbubble complex was established.

A recent study regarding ultrasound-responsive prodrugs (prodrugs composites) has been reported by Gao et al., 2019 [216]. Initially C_9_F_17_-poly{N-[N’-(2-aminoethyl)]-aspartamide}(diethylene triamine)] denoted as C_9_F_17_-PAsp(DET) was synthesized by ring opening polymerization from C_9_F_17_-NH_2_.The resultant compound was then converted into C_9_F_17_-PAsp(DET)-FITC by interacting with fluorescence iso-thiocyanate and then subjected to lyophilization to achieve lyophilized C_9_F_17_-PAsp(DET)-FITC. After synthesis of the abovementioned conjugate, cis-aconityl-doxorubicin was synthesized (as mentioned earlier by Du et al., 2013). The author reported the development of ternary per-fluoro-pentane/C_9_F_17_-PAsp(DET)/cis-aconityl-doxorubicin nanodroplets and revealed the ultrasound-assisted in vitro cytotoxicity of tumor cells. The per-fluoro-pentane was used as an ultrasound-responsive agent, which showed enhanced sensitization to the ultrasound and overall optimum cellular uptake and tumor cell cytotoxicity was achieved. There has been reported very limited studies on ultrasound-responsive prodrugs for cancer therapy. Therefore, this area could be considered as a research gap for novel and advanced cancer research in the future. In addition, obstacles regarding handling, tumor specificity and achievement of optimum cytotoxicity could also be resolved in the modern arena of research.

##### Light-Responsive Prodrugs

Light-responsive prodrugs are used to elicit toxicity at the tumor site upon using a specific source, wavelength and magnitude of light, in conjunction with a light-sensitive/activate-able drug. In contrast to the photothermal NIR-based prodrugs, light-responsive prodrugs do not cause hyperthermia with the application of light. In addition, light-responsive prodrugs are based on the use of anticancer drugs or agents which are responsive and activated by the applied light (photosensitizers) in conjunction with the specific light sources (based on wavelength and color) followed by the destruction of tumor cells. This type of prodrug-based therapy is known as photosensitizer-based photodynamic therapy [235]. Bourre et al., 2008 [217], reported the development of short peptide-based ALA derivatives as naturally occurring porphyrins-based photosensitizers commonly known as protoporphyrin. ALA is endogenously synthesized in heme synthesis pathways and is associated with quick clearance from the body. Due to the abovementioned reason, the exogenous ALA derivatives are used as light-responsive prodrug for tumor-specific targeting. Juzeniene et al., 2007 [235], also reported that the aforementioned photosensitizer target itself at the tumor site followed by the destruction of tumor cells (mostly skin cancer cells). After successful accumulation at the tumor site, usually, the red light is used for the activation of protoporphyrin, which in turn causes reactive oxygen species (ROS) production and cancer cells-specific necrosis. It has been reported that three different compounds of short peptide-based ALA derivatives were synthesized in which compound 1 (n-Acetyl-L-phenylalanyl-n(benzyloxycarbonyl)-5-aminolaevulinic acid methyl ester) was the most effective regarding photosensitization, lipophilicity and tumor-specific cytotoxicity. In addition, it was also reported that short peptide-based ALA derivatives were synthesized to enhance intracellular entry across the biological membrane (active targeting) through improved lipophilicity and selectivity towards cancer cells through esterases. Hossion et al., 2013 [218], reported the use of active constituent of combretastatin A-4 phosphate (CA-4), an active metabolite of irinotecan (SN-38) and coumarin-based prodrugs (D) in conjunction with a deactivated photosensitizer (dps) and amino-acrylate cross linker (L) for theranostic application. The aforementioned cross linker (L) also acted as an intracellular esterase-responsive and singlet oxygen-responsive photosensitizer and was synthesized by click chemistry in a conjugated form with the drug. The author reported that the drug cross-linker-deactivated photosensitizer (D-L-dps) complex, caused the activation of deactivated photosensitizer through intracellular esterases. In addition, the irradiation of light (combination of visible light = 540 nm and NIR) caused the release of singlet oxygen. The ultimate effects were cleavage of cross linker and achievement of in vitro cytotoxic effects through photo-unclick chemistry. A similar concept has also been reported by Lin et al., 2016 and Zhang et al., 2020 for the ROS responsive systems [236,237]. Mari et al., 2015 [238], reported the development of several compounds in the form of triplets in the conjugated form with ruthenium for photothermal active targeting-based cytotoxicity. The objective was achieved through ruthenium-polypyridyl complex as an antineoplastic agent, Photolabile protecting group (PLPG) for photoactivation of prodrug and a targeting peptide for high uptake and overexpression of onco-cyto-receptors. The authors reported the use of ruthenium-(Ligands)- dipyrido[3,2-*a*:2,3-*c*]phenazine)-CH_2_NH_2_ as Ru-L-dppz structure for the initiation of the study. Initially, three different types of ligands were selected namely 2,2-bipyridine (A), 1,10-phenanthroline (B) and 2,2-dipyridylamine (C) as Ru-A, Ru-B and Ru-C. It was observed that Ru-A and Ru-C showed slight cytotoxicity while Ru-B showed no cytotoxicity. The PLPG as a photoactivatable moiety nuclear localized signal (NLS) as genetic material targeting and the nuclear localization signaling moiety were initially conjugated to Ru-A, Ru-B and Ru-C and to achieve Ru-A-PLPG-NLS, Ru-B-PLPG-NLS and Ru-C-PLPG-NLS. It was reported that Ru-A-PLPG-NLS and Ru-B-PLPG-NLS showed cytotoxicity but Ru-C-PLPG-NLS showed no toxicity. In the next step, The PLPG was conjugated to Ru-A, Ru-B and Ru-C and peptide bombesin (component for overexpression of gastrin-releasing peptide) to achieve Ru-A-PLPG-bombesin, Ru-B-PLPG-bombesin and Ru-C-PLPG-bombesin. The results showed that Ru-A-PLPG-bombesin and Ru-B-PLPG-bombesin showed higher cytoplasmic and nuclear cytotoxicity in HeLa cells, but the use of a light source in MRC-5 cells did not show significant photo-toxicity or cytotoxicity. The author reported that the reason might be associated with the wavelength of light, used in the study. Liu et al., 2016 [219], also reported the development of an anticancer drug (gemcitabine) conjugated with fluorescence/photo sensitizer (mesotetraphenylporphyrin) and thioketal as singlet oxygen-cleavable cross linker. Contrary to previous studies, meso-tetra-phenyl-porphyrin (mTPP) was used for the production of singlet oxygen, thioketal cross linker (L) as singlet oxygen-cleavable cross linker and gemcitabine (GEM) in conjugated form were used for cascade chemotherapy. In brief, initially the m-TPP and thioketal crosslinker were synthesized followed by conjugation of mTPP with thioketal cross linker and subsequent conjugation of mTPP-thioketal cross linker conjugate with gemcitabine. Finally, the encapsulation of mTPP-L-GEM was achieved within PEG_2000_-PLA_2000_ for the achievement of mTPP-L-GEM PEG_2000_-PLA_2000_ micelles. The authors reported that encapsulation of PEG_2000_-PLA_2000_ was done to facilitate targeting through the micelles, and the results showed successful achievement of singlet oxygen (as ROS) production by mTPP through irradiation of light (658 nm). This was followed by cleavage of thioketal crosslinker, the achievement of the cascaded release of gemcitabine, enhanced tumor localization, better cancer cells in vitro and in vivo uptake and cancer cells-specific apoptosis, respectively. Thapa et al., 2017, reported the development of far-red light-responsive PEGylated folic acid (FA-PEG_n_)-conjugated paclitaxel (PTX)-based prodrug micelles in conjugation with phthalocyanine (Pc). The Pc was used as a photosensitizer and singlet oxygen-responsive cross linker. The irradiation of far-red light was done to achieve tumor-specific active targeting of the prodrug micelles. The authors reported that paclitaxel as a highly lipophilic drug is associated with non-specific cytotoxicity; therefore, PEGylated folic acid-conjugated paclitaxel (as discussed earlier by Talelli et al., 2011 and Zhang et al., 2016) was used. Initially, the Pc-L-PTX (compound 3) was synthesized by already synthesized compound 1 and 2 followed by conversion into Pc-L-PTX-acid (compound 4) and subsequent conversion of compound 3 (Pc-L-PTX) into FA-Pc-L-PTX (compound 5) and likewise conversion of compound 4 into FA-PEG_n_-Pc-L-PTX (*n* = 2000 and 5000). The author reported that upon entry of the developed prodrug through folate receptors overexpression (active targeting), laser irradiation of far-red light (690 nm) caused the photo-activation of cross linker, releases of singlet oxygen, cleavage of aminoacrylate cross linker and cancer cell-specific apoptosis. In another study, the synthesis of photosensitive CA-4-based prodrug, in conjunction with the protoporphyrin as photosensitizer (as mentioned earlier by Bourre et al., 2008) for successful photo-unclick chemistry-assisted visible light-based mitochondrial toxicity was achieved (as mentioned earlier by Hossion et al., 2013). In these studies, active targeting-based cytotoxicity was reported, though the description of the mechanism was not elucidated [220,239]. Yang et al., 2018 [221], reported the development of redox-sensitive pyropheophorbide a (PPa)-entrapped cabazitaxel-oleic acid-single thioether/selenoether-conjugated self-assembly ROS-activated prodrug nanoparticles. The cabazitaxel-single thioether-oleic acid was denoted as CTX-S-OA, whereas, cabazitaxel-selenoether-oleic acid was represented as CTX-Se-OA. The prodrug nanoparticles through one-step nanoprecipitation method were reported using DSPE-PEG_2000_ for PEGylation, cabazitaxel as anti-tumor agent, single thioether and selenoether as ROS-responsive cross linkers and PPa was used as photosensitizer. In brief, cabazitaxel (CTX) was initially conjugated with oleic acid (OA) followed by conjugation with single thioether (S) and selenoether (Se) to achieve CTX-S-OA and CTX-Se-OA prodrugs. The synthesized prodrugs were then incorporated into DSPE-PEG_2000_ solution, followed by the incorporation of PPa solution and subsequent removal of organic solvents to achieve PPa-loaded CTX-S/Se-OA⸞DSPE-PEG_2000_ prodrug nanoparticles. The prodrugs nanoparticles in conjunction with 660 nm laser irradiation both in vitro and in vivo were used and tumor-specific localization, active targeting through endocytosis-mediated uptake and enhanced ROS-activated photo-chemotherapy was achieved. Phua et al., 2019 [240], reported the activated camptothecin-photosensitive and singlet oxygen-sensitive β-cyclodextrin-modified hyaluronic acid-based prodrug nanoparticles. Briefly, camptothecin prodrug was synthesized by a series of five reactions followed by synthesis of the activated photosensitizer. Subsequently, β-cyclodextrin-modified hyaluronic acid was synthesized (as mentioned earlier by Wang et al., 2015). The aforementioned components were then conjugated as camptothecin-conjugated β-cyclodextrin-modified hyaluronic acid and photosensitizer through a singlet oxygen-cleavable cross linker, followed by self-assembling to achieve HA-PS-CPT nanoparticles. In addition, the achievement of cleavage of cross linker through singlet oxygen produced by photosensitizer after irradiation of 660 nm light and in vitro and in vivo cancer cells-specific apoptosis was successfully achieved. Chen et al., 2019 [20], developed an NIR-based light-sensitive and ROS-cleavable prodrug for photodynamic therapy through host–guest strategy. Initially, PEG-PGA-β-CD was synthesized through conjugation of β-cyclodextrin with polyethylene glycol and poly-L-glutamate, followed by synthesis of Adamantane-modified aza-BODIPY (Ada-BODIPY) and Adamantane-modified paclitaxel (Ada-PTX). In this case, the adamantane linkages acted as ROS-cleavable linkers, BODIPY as photosensitizer and PTX as an antitumor agent. In addition, PEG-PGA-β-CD was used as a host molecule, and Ada-BODIPY, Ada-PTX as guest molecules for the achievement of host–guest complex-based self-assembled nanoparticles was reported. After irradiation of NIR (660 nm), the ROS production was followed by the cleavage of adamantane linkages and achievement of in vitro and in vivo cascaded paclitaxel-based chemotherapy for tumor-specific cytotoxicity through passive targeting was established.

A recent study regarding light-responsive prodrugs has been reported by Zhang et al., (2020). In this study, the authors investigated the dual active and passive targeting of tumors through a combinatorial strategy of chemotherapy and gene therapy. This was demonstrated through PEGylated hyaluronic acid-based light activation in parallel with RNA interference of Pt(IV) prodrug backboned polymeric (CNPPtCP/si(c-fos) nanoparticles for platinum-resistant ovarian cancer. The aforementioned prodrug nanoparticles contained anticancer agent Pt(IV) and the cancer gene (cfos) for overexpression of CD44 receptors. The PEGylated hyaluronic acid-based nanoparticles were fabricated and results showed that upon irradiation of blue light (430 nm) the dual chemotherapy and gene therapy was achieved. The study provided a piece of evidence regarding enhanced cellular uptake and tumor-specific apoptosis through active targeting. In addition, the current concept of dual active and passive tumor targeting by dual cancer and gene therapy via light-responsive prodrugs or multiple stimuli-responsive prodrugs could be considered as a future prospect for the management of various types of malignancies [241].

##### pH-Responsive Prodrugs

The pH-responsive prodrugs are prodrugs that release the drug only at a specific pH range. Wang et al., 2014 [223], reported the development of polymeric prodrugs which self-assembled into micelles. This was done by the development of PMAC (poly(5-methyl-5-allyloxycarbonyl-1,3-dioxan-2-one) through ring opening polymerization, functionalization of PMAC (thiol-PMAC), to achieve PMAC-graft-(ADPC-co-Mal-DOX) by interacting with Mal-DOX (6-maleimidocaproyl doxorubicin) and ADPC (12-acryloyloxy dodecyl phosphorylcholine) through click chemistry. It has been reported that doxorubicin was released at the acidic pH (tumor site) through passive targeting by the cleavage of hydrazone bonds to which doxorubicin was connected as a prodrug [222]. Likewise, Wang et al., 2016, reported polyethyleneimine-based aforementioned conjugate (ADPC-PEI-Mal-DOX) for passive targeting. In contrast, in another study, Wang et al., reported dual pH and hydrogen peroxide-responsive prodrug-based stimuli-responsive targeting. By contrast, Wang et al., (2015) and Yu et al., (2017) reported dual pH and reduction-sensitive prodrugs for extravasation through EPR effect [222,242,243]. Xing and Yan (2014) [244] reported the synthesis of eight different types of polymers/copolymers through ring opening polymerization and RAFT polymerization, followed by conjugation of near infra-red probe and doxorubicin. In short, pH-responsive polymeric prodrugs for in vitro tumor targeting were achieved. Zhang et al., (2016), reported the development of pH-responsive nanoparticles containing prodrugs as the combination of methoxy-poly (ethylene glycol)-aldehyde and doxorubicin through Schiff base reaction. The development of prodrug was achieved through the series of reactions including attachment of amino group of doxorubicin with the aldehyde mPEG-CHO group. The self-assembly into PEG-DOX in an aqueous environment at pH 7.4, followed by incorporation of hydrophobic curcumin, resulted in of the formation of PEG-DOX-Cu nanoparticles. In the acidic pH of the tumor, both curcumin and doxorubicin were released due to the cleavage of Schiff’s base and passive targeting of the tumor. Likewise, in another study, the authors reported the development of dual reduction and pH-responsive prodrug nanogels (pH-responsive prodrug and core nanogel) for passive targeting using doxorubicin as a model anticancer drug [245,246]. Raveendran et al., 2016, also reported pH-responsive passive targeting of cancer cells through curcumin and dextran conjugate [226]. Ma et al., 2016 [247], reported the development and passive targeting of pluronic F127-chitosan-doxorubicin in combination with paclitaxel. It has been reported that pluronic F127 was grafted with chitosan followed by conjugation with doxorubicin and subsequent physical encapsulation of paclitaxel inside the abovementioned conjugate. The developed conjugate was able to achieve tumor-specific localization through EPR effect. He et al., 2019 [224], reported the development of mPEG-b-PLA-g-DOX by strain-promoted alkyne-azide cyloaddition technique for in vitro cancer cell-specific targeting through extravasation from the leaky vasculature around the tumor (passive targeting). The mPEG-b-PLA-g-doxorubicin was synthesized by a series of reactions including mPEG-b-norbornene functional PLA, mPEG-b-PLA-g-COOH, Cyclooctyne doxorubicin and mPEG-b-PLA-g-DOX synthesis. The in vitro intracellular delivery of doxorubicin was also reported. Du et al., 2013 [225], reported the dual active and passive targeting of a prodrug at the tumor site by using the pH-responsive cross linking agent in the conjugated form with doxorubicin, bovine serum albumin and folic acid. Initially, the cis-aconitic anhydride-doxorubicin complex was formed through conjugation of doxorubicin with cis-aconitic anhydride (pH-responsive cross linker), followed by mixing of bovine serum albumin with the aforementioned mixture, and subsequent conjugation with folic acid to achieve BSA-CA-DOX-FA. The aforementioned complex has been shown to be successful at passive drug targeting by cleavage of cis-acotinic anhydride and release of doxorubicin at tumor site due to the acidic micro-environment of the tumor. In addition, entry of drug within the cell was also achieved through active targeting by overexpression of folate receptors and apoptosis. Li et al., 2014 [248], reported the dual active and passive pH-triggered targeting for theranostic application. The authors reported the achievement and monitoring of targeting through the initial synthesis of 6-maleimidocaproyl hydrazine derivatives of doxorubicin followed by conjugation with a coumarin-grafted peptide through thiol maleimide click reaction. The fabricated compound includes peptide for overexpression of tumor cell receptors (alpha and beta 3), coumarin for monitoring of change in intensity of fluorescence (as diagnostic agent) and doxorubicin for tumor-specific localization and cancer cell necrosis. Likewise, Luo et al., 2017 [215], reported dual active and passive targeting of doxorubicin through a pH- and ultrasound-responsive folate-conjugated doxorubicin prodrug microbubble complex for theranostic application. Moreover, Wang et al., 2018 [249], in their review article stated their experience that drug release from pH-responsive prodrugs could be done through addition or removal of protons from the polymer(s), deprivation of amphiphilic block from polymer(s), increase in hydrophilicity of polymer(s) and/or cleavage of acid-labile bond between the drug and polymer.

Jin et al., 2020, has recently exploited the pH-sensitive prodrug-based technology for effective synergistic effects of cisplatin and doxorubicin in treating lung cancer [250]. The developed formulations showed an enhanced physicochemical stability, in vitro cytotoxicity and in vivo anti-tumor efficiency and bio-distribution. Moreover, the above study on pH-sensitive prodrugs could be extended to multiple stimuli-responsive prodrugs associated with multiple therapeutic agents for active and passive targeting of tumors through dual immune therapy and chemo-therapy/chemo-phototherapy. In the future, the pH-sensitive prodrugs should be subjected to human clinical trials to depict the safety and efficacy of the pH-responsive prodrugs against a variety of malignancies and various other biomedical applications.

#### 2.2.4. Stimuli-Responsive Carriers/Polymers

**Figure 6:**
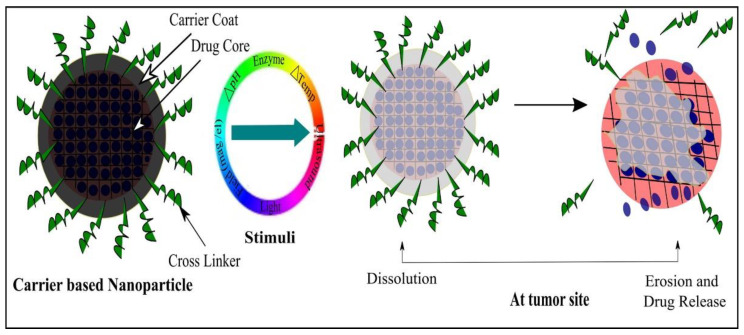
Stimuli-Responsive Carrier/Polymer-based Nanoparticle for Cancer Chemotherapy.

Targeted delivery approaches are of prime importance to deliver the drug to the target site. Carrier-based drug delivery systems are sometimes designed for the achievement of optimum therapeutic effects at the particular site by enhancing the drug release characteristics and targeting through the use of a carrier in conjunction with therapeutic moieties. These carriers have the stimulus-responsive effects by various external and internal stimuli; the stimuli-responsive carriers are therefore known as “smart biomaterials”. These stimuli-responsive carriers may be dissolved in an aqueous solution, or adsorbed or chemically grafted at the aqueous–solid interface. They might alternatively be chemically cross-linked, or attached through hydrogen bonding (with excipients or drug), or physically (as in the case of hydrogels) [251]. Stimuli-responsive carriers are mainly composed of moieties that are sensitive to various stimuli such as temperature, pH, light, magnetic field or electric field (Table 7). Temperature, light and magnetic field, and ultrasound are applied to the areas of the body as external stimuli whereas, pH and enzyme are internal body stimuli that are present within the microenvironment of the target tissue. The various stimuli-responsive carriers could be useful for targeted or controlled drug release (Figure 6) [190]. This review focuses on the stimuli responsiveness of the carriers towards the tumor microenvironment, such as:

- Temperature-responsive carriers/polymers;
- Magnetic/electric field-responsive carriers/polymers;
- Ultrasound-responsive carriers/polymers;
- Light-responsive carriers/polymers;
- pH-responsive carriers/polymers;
- Enzyme-responsive carriers/polymers.

##### Temperature-Responsive Carriers/Polymers

Temperature-responsive carriers, also referred to as thermoresponsive carriers/polymers, are drug delivery systems that release the drug within a specific temperature range. Polymeric thermoresponsive carriers showed improved aqueous solubility at specific temperatures/temperature ranges (lower critical solution temperature) (LCST) and above this temperature/temperature range, the carriers/polymers are insoluble. Poly (*N*-isopropylacylamide) (PNIPAAm) is a thermoresponsive polymer that exhibits an LCST of 32 °C. At this temperature, the PNIPAAm shows hydrophilic characteristics, whereas, above this temperature, it became hydrophobic. Thus, the carrier, through its thermosensitivity, is destabilized at the cancer temperature (hyperthermia) through polymeric erosion or disruption followed by the release of drug after passive targeting at the tumor site [274,275].

Liu et al., 2005 [252], prepared thermosensitive micelles of P(PNIPAAAm-*co*-DMAAm)-*b*-PLGA (Poly (*N*-isopropylacrylamide-*co*-*N*, *N*-dimethylacrylamide)-*b*-poly(D,L-Lactide-*co*-glycolide)) loaded with doxorubicin. Thermosensitive block co-polymers were successfully synthesized and then doxorubicin was incorporated into micelles. The LCST of the micelles was 39 °C in phosphate buffer. Below LCST, mild release of doxorubicin was achieved from the micelles, whereas, the doxorubicin-loaded micelles showed enhanced cancer cell-specific cytotoxicity above the LCST through passive targeting. Liu et al., 2013 [253], prepared thermoresponsive PNIPAAm-based hydrogels by free radical micellar copolymerization method. Below 32 °C the hydrogels were hydrated, swollen and are regarded as hydrophilic in nature, whereas, above that temperature, the hydrogels showed dehydrated and hydrophobic nature due to breakage of its cross-linking network. Rejinold et al., 2011 [254], prepared thermoresponsive Poly (*N*-vinyl caprolactam)-based chitosan nanoparticles for 5-fluorouracil by ionic cross-linking method. The Poly (*N*-vinyl caprolactam) showed LCST at 38 °C and in addition, the thermoresponsive chitosan-based nanoparticles due to the presence of Poly (*N*-vinyl caprolactam) showed temperature-induced phase transition above LCST, enhanced in vitro drug release, cancer cells-specific passive targeting-based cytotoxicity and apoptosis. Khan et al., 2019 [267], developed dual temperature- and pH-responsive 5-fluorouracil-loaded Poly(*N*-isopropylacrylamide)/carboxymethyl chitosan injectable hydrogels. The injectable hydrogels were prepared by cross-linking method and evaluated for various parameters. The developed system showed enhanced drug release at hyperthermia, good morphology, improved cytotoxicity through passive targeting, apoptosis and hydrophobicity of formulations above 32 °C.

The thermoresponsive carriers have been recently exploited for delivery of doxorubicin. Shin et al., 2020 [276], fabricated cancer targeted near-infrared and thermoresponsive PNIPAM-pyrrole nanocomposites for the delivery of doxorubicin. Folic acid, as a targeting ligand was conjugated in the PNIPAM network for active targeting of the breast cancer cells using doxorubicin as anticancer agent. The enhanced anti-cancer efficacy was due to specific cellular uptake of nanocomposites in folate-receptor-mediated endocytosis and doxorubicin release from PNIPAM network via near infrared and thermoresponsive effects. The recent study could be potentially considered for the delivery of multi-stimuli-responsive drug delivery carrier for synergistic therapeutic approaches towards different types of cancer with reduced side-effects.

##### Magnetic/Electric Field-Responsive Carriers/Polymers

Magnetic field-responsive carriers/polymers may respond to magnetic fields or contrast agents. They may exists in solution as free chains, immobilized to surface molecules, or be cross-linked within networks (e.g., norbornene based co-polymers) [277], [278]. Mostly in stimuli-responsive polymers, the cascaded response of many polymers could be observed which may be overcome by magnetic-responsive carriers, as these carriers are convenient and show enhanced sensitization and quick cytotoxicity in the presence of magnetic/electric field or contrast agents [279]. Mukherjee et al., 2016 [255], synthesized a norbornene-based co-polymer for the delivery of biotin and doxorubicin for theranostic application. The norbornene-based co-polymer for the delivery of biotin (MRI contrast agent) and doxorubicin (anticancer agent) is considered for potential magnetic resonance imaging for cancer diagnosis and passive targeting of cancer. The block co-polymers were prepared (ring opening polymerization technique) and revealed enhanced normal cell viability, improved cancer cells cytotoxicity and enhanced stimulus-based drug release of norbornene-based doxorubicin and biotin-loaded nanocarriers as MRI agent and anticancer drug delivery applications. M. Zrinyi (2000) [280] reported the magnetic field effects on the mobility of the gels. In the presence of uniform magnetic field, the gel beads will form a straight chain structure, whereas, in non-uniform magnetic field, the gel beads form aggregate-like structures, which confirm the responsiveness of polymers towards the magnetic field. Zha et al., 2012 [256], prepared oxanorbornene-based cobalt containing di-block co-polymers by ring opening polymerization technique. The polymer containing thin films were used, containing room temperature ferromagnetic materials that retained the block co-polymer structural morphology with the treatment of heat. The ordering of these block co-polymers was of prime importance for the ferromagnetic behavior.

Magnetic mesoporous silica nanocomposites have been found to be very effective for breast cancer as dual chemotherapy and photodynamic therapy [281]. These nanocomposites have excellent cellular uptake, circumvent the drug resistance and induced apoptosis in cancer cell lines (MCF-7/ADR cells). The synthesis of magnetic field-responsive carriers and multi-therapy is supposed to be an attractive research direction in the future. In addition, complicated and programmed responsive behavior upon a magnetic field deserves more scientific attention to develop multiple (dual or triple) therapy-based drug delivery systems utilizing magnetic-responsive carriers.

##### Ultrasound-Responsive Carriers/Polymers

The ultrasound-responsive carriers/polymers use ultra-high-frequency waves generated by mechanical oscillations and are used for different diagnostic or treatment applications. Ultrasound is used in different fields such as in ultrasound devices, kidney stone treatment, magnetic resonance and imaging [277]. Recent studies suggests that ultrasound-responsive carrier systems are capable of enhancing solubility, extravasation (EPR) and targeting the incorporated drug to the cancer tissue, with subsequent improvement of drug uptake by cancer cells. This occurs as a result of enhanced drug release at the tumor site and/or increased cell membrane permeability in response to the ultrasound waves [120,282]. Rapoport (2004) [262] formulated ultrasound-responsive Pluronic P-105 (block co-polymer) micelles for the delivery of doxorubicin. Pluronic acid is tri-block cop-polymer with the molecular formula of Poly (ethylene oxide)-Co-Poly (prolylene oxide)-Co-Poly (ethylene oxide), with lipophilic Poly(prolylene oxide), (PPO). The doxorubicin micelles prepared were then investigated for drug release. The degree of drug release was then measured when doxorubicin was transferred from lipophilic environment of micelle cores to the hydrophilic environment with ultrasound. The study suggests that the micelles could effectively deliver, target and localize doxorubicin to the tumor site through the use of ultrasound-responsive carriers. Rapoport et al., 2009 [261], prepared ultrasound-responsive microbubbles for the delivery of paclitaxel. The nanoemulsions formulated were then converted into microbubbles at the target tumor site, by applying directed ultrasound at the tumor.

Both the pH-sensitive hollow mesoporous organo-silica and gemcitabine nanoparticles have been found with strong anticancer potential [283]. The formulated gemcitabine nanoparticles are expected to provide an alternative way of improving anti-tumor efficacy by the downregulation of the extra-cellular matrix and offer a passive-targeted therapy for cancer treatment. With the therapeutic efficacy and low systemic toxicity of hollow mesoporous organo-silica nanoparticles in the SW1990/PSCs pancreatic tumor-bearing mice, the nanoparticulate drug delivery carrier showed great potential to deliver nanomedicine with controlled release for effective cancer therapy. The combination therapy may be effective for theranostic applications of anti-cancer therapy to deliver the chemotherapeutic effect at the targeted site with minimal side-effects.

##### Light-Responsive Carriers/Polymers

Light-responsive carriers/polymers are based on applying external light stimuli on drug carriers that have inherent properties to change their characteristics in response to certain types of light. Thus, light-responsive carriers are capable of releasing the drug at a suitable light wavelength, and intensity. These light-responsive carriers/polymers can alter certain properties in response to light. Examples of light-responsive polymers are azo-benzenes, cinnamon derivatives and triphenylmethane leuco derivatives. Hence, carriers are treated with photoresponsive functional groups or fillers that would make the overall structure of the carrier highly responsive. For example, azo-benzenes exists in cis and trans-isomeric forms, and can be switched to one another by exposure of light of suitable wavelength (e.g., cinnamon derivatives have the potential to replace the thermo-reversible physical cross-linking, whereas, triphenylmethane leuco derivatives dissociates into respective ion pairs (upon exposure to UV light). Jiang et al., 2006 [263], reported that when cinnamic acid (cinnamon derivative) is exposed to UV-light with the wavelength of less than 260 nm, the double bond of cinnamic acid dimerizes with the adjacent cinnamic acid to form the cyclo-butane rings. The cyclo-butane rings are capable of eliciting cytotoxic effects at the target site through EPR effect, and in the contrary, reversible photo cleavage upon exposure to UV light of more than 260 nm. Similarly, other derivatives of cinnamon such as cinnamylidene acetic acid also dimerizes at the wavelength of more than 300 nm [264,265].

Recently, Wang, Y., et al., 2019 [224], have synthesized photothermal responsive magnetic mesoporous silica nanocomposites for non-small cell lung cancer (NSCLC). Using NSCLC model, multifunctional nanoplatform aptamer-based gold silica platform for the targeted therapeutic activity has been successfully designed for enhanced NSCLC therapy. Furthermore, the magnetic field and photothermal synergistic effects may also play an important role in the achievement of effective therapeutic outcomes than chemotherapy alone or conventional drug delivery strategies.

##### pH-Responsive Carriers/Polymers

The normal blood pH range is 7.4–7.5, but the pH of the tumor site is acidic in the range of 5.0–6.5. The decrease in the pH at the tumor site is attributed to the decrease in oxygen supply to tumor vessels resulting in ischemic condition, causing the interstitial fluid to become acidic [284,285]. The pH-responsive carriers/polymers are designed to provide the pH-responsive sheddable shell consisting of multiple layers, such that one of the layers is a neutral layer which is shed in response to the acidic tumor microenvironment. In addition to the mentioned neutral layer, they also contain the charged nanoparticles surface/layer (usually cationic) with the capability of facilitating neoplasia-specific cellular uptake due to the attraction as the cancer cell surfaces usually have a negative charge [286]. Liu et al., 2009 [269], developed pH-responsive polymer-based hydrogels and revealed that the increase in the pH caused the ionization of poly acrylic acid (PAA), whereas, at lower pH the chains of PAA (at carboxyl group) were protonated and caused the in vitro destabilization of hydrogels and remained stable at pH 7.4. Thus, the evidence for passive targeted cancer therapy was achieved followed by enhanced cancer-specific cytotoxicity and apoptosis. Ding et al., 2018, [287] synthesized pH-sensitive coiled-coil peptide-crosslinked hyaluronic acid nanogels for targeted intracellular delivery to CD44 receptors for overexpressing MCF-7 breast cancer cells. The anti-cancer proteins such as granzyme-B, saporin and cytochrome-C are used as smart approach for cancer protein therapy. Flow cytometry and confocal studies showed that protein-loaded hyaluronic acid nanogels are efficiently taken up by MCF-7 cells through a receptor-mediated pathway. The coiled-coil peptide-cross-linked hyaluronic acid nanogels have appeared as a simple and multi-functional platform for good intracellular protein therapy. Likewise, Lin et al., 2018, [288] fabricated doxorubicin-loaded zwitterionic arginyl-glycyl-aspartic acid conjugated polypeptide for pH-induced disintegration and enzyme-induced degradation of the nano-formulation. The biodegradable zwitterionic-modified doxorubicin-loaded polypeptide vesicles were prepared by emulsion solvent evaporation method. The prepared vesicles showed excellent stability, enhanced intracellular drug release (pH and enzyme-responsive), lower systemic toxicity and enhanced tumor accumulation.

Transdermal siRNA delivery by pH-responsive micelles has been successfully achieved for melanoma therapy [289]. This study provides the design of pH-switchable cationic micelles as transdermal gene delivery nanoplatform with targeting effects and pH-triggered release. The combination of gene therapy and pH-responsive therapy plays an imperative role and has the potential to cure cancer as a novel drug carrier. Moreover, the combination of these smart nanocarriers with efficient therapeutic genes is expected to promote the application of dual chemotherapy and gene therapy in humans with improved safety and efficacy.

##### Enzyme-Responsive Carriers/Polymers

Enzyme-responsive carriers/polymers are made of bioresponsive nanomaterials that can respond to the catalytic property of an enzyme. Enzyme-responsive carriers are unique in their ability to be cleaved by exposure to target enzymes, or to the bioactive agent(s) for its optimum subsequent diagnostic or therapeutic theranostic activity. The enzymes utilized are commonly hydrolases, proteases, lipases or glycosidases [163,249].

Ferguson et al., 2009 [270], prepared dextran-phospholipase A_2_ (dextran-PLA_2_) polymeric conjugates for the delivery of α-amylase. The hydrolytic activity of the enzyme was enhanced by α-amylase when exposed to dextran-PLA_2_ polymeric conjugates. The PLA_2_ are esterases in nature that can catalyze the hydrolysis of phospholipids (breakage of acyl bond) to release the lysophospholipid and free fatty acid. The dextran-PLA_2_ polymeric conjugates showed potential activity as novel anti-cancer agents. Vicent et al., 2005, [271] prepared *N*-(2-hydroxypropyl) methacrylamide (HPMA) co-polymeric conjugates for the delivery of doxorubicin. The polymeric conjugates synthesized showed combined chemotherapy and endocrine effects. The chemotherapy effects were confirmed by cellular uptake of the conjugate via endocytosis. Roberto de la Rica (2011) [272] developed perthiolated β-cyclodextrin gold nanoparticles as ultra-responsive enzyme sensors for the delivery of peroxidase. Peroxidase is widely used as an enzymatic tool for enzyme-linked immunoassays. The sensitivity of gold nanoparticle biosensors was enhanced by assembling their building blocks through supra-molecular multivalent interactions.

Based on the recent studies, Barve et al., 2020 [290], reported enzyme-responsive polymeric micelles for the delivery of cabazitaxel for prostate cancer. The polymeric micelles demonstrated better inhibition of tumor growth than free cabazitaxel in mice bearing prostate cancer xenografts. Gene-directed enzyme-responsive carriers are considered to revolutionize the goals of chemotherapy for the treatment of various types of cancer.

## 3. Molecular Dynamics (MD) Simulations Associated with Stimuli-Based Tumor Targeting

The rational release of drugs from multi-functional nanoparticles can be precisely predicted by the use of multiscale computational modeling. These mechanistic models can better provide the knowledge regarding the fundamental interactions between drug, carriers and patient. However, this multiscale computational modeling is in its early days for traditional drug delivery. For emerging nanotechnology systems for drug delivery, a reliable in silico prediction is needed. In the past, the drug development stages have shown that ~39% of failures are due to poor pharmacokinetics and ~11% are due to toxicity. So, the in silico prediction of the pharmacokinetics of potential new entities in nascent stages of development has become a critical area of research nowadays. Molecular interaction between nano/micro-scale and drug, vehicles, GIT and target site requires computational chemistry approaches such as molecular dynamics (MD) and mesoscale modeling. Stochastic approaches are employed to predict the effect of variance that arises from biological sources both at macro and micro/nano levels [291,292].

**Figure 7:**
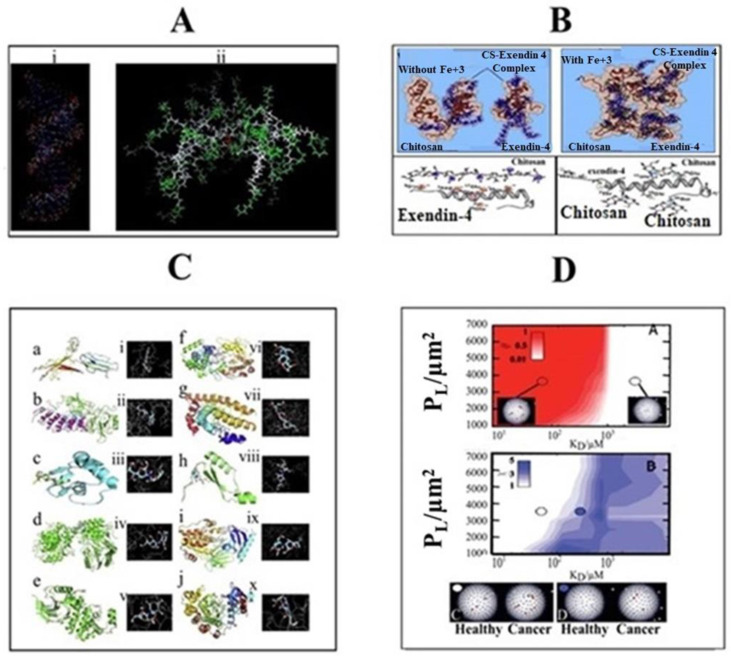
Molecular Dynamic Simulation of (**A**) siRNA-based dendrimers, (**B**) interaction between 4 20-mer chitosan (CS) and 9 exendin-4 molecules in the absence/presence of Fe3+ at pH 6.5, (**C**) docking scheme 2D model representing the affinity and (**D**) selectivity of nanoparticles towards healthy and cancerous cells (adopted with Copyright permission).

In stimuli-responsive systems, the use of molecular dynamics simulations is an emerging field with great potential. The experimental findings can be better simulated by the use of atomistic modeling, and particularly all-atom molecular dynamics (MD) simulations [293]. This high molecular simulation will provide the unique detail about the polymer structure, its interaction with solvents and structural changes that occurred with stimuli. Using MD simulations, the effect of pH not only provides insight into any structural changes in dendrimers but also the possible interaction of the dendrimers with other molecular targets such as siRNA (Figure 7A) [294,295]. Similarly, carter et al., 2014 [33], identified a novel light-absorbing monomer by using molecular dynamics simulations which give rise to a more stable porphyrin bilayer across liposomes. Thus, light-induced membrane permeation was enabled with inclusion of 10 molar % porphyrin-phospholipid within liposome formulations. Nguyen et al., 2011 [296], performed MD simulations in a full-atom model to provide a clear perception about possible interaction between chitosan and exendin-4 in the presence/absence of Fe^3+^. In that study, nine exendin-4 and four 20-mer chitosan molecules were used in the MD simulation at pH 6.5, in which the ratio of deprotonated (–NH_2_) and protonated (–NH_3_^+^) amine groups on chitosan was about 1:1. The pI (phosphoinositide) of exendin-4, containing five glutamic acids (Glu) and one aspartic acid (Asp), is around 4.2. The result showed that in the absence of Fe^3+^, an electrostatic interaction was formed between two sets of complexes, each containing two chitosan and three exendin-4 molecules, while three exendin-4 molecules were left alone. In contrast, in the presence of iron, a single complex consisting of four chitosan and nine exendin-4 molecules was found due to coordinate-covalent bonds in the locality of nitrogen as well as carboxylic, glycosidic, and hydroxyl oxygen atoms. Therefore, apart from electrostatic interactions, in the presence of Fe^3+^, coordinate-covalent bonds were formed between chitosan and exendin-4, which significantly increased the drug loading in the preparation of test nanoparticles (Figure 7B). Sekar et al., 2018 [297], performed the in silico molecular docking studies to examine the possible interaction of chitosan ascorbate nanoparticles (CANs) with significant targets of cervical cancer. Eighty sets of docked complexes were generated by docking of each enzyme with chitosan ascorbate nanoparticles (Figure 7C). The highest and lowest binding energy for the docking of CANs with 10 enzymes were determined. These different proteins were associated with different cell signaling pathways, causing rapid cell growth, neo-vascularization, abnormal apoptosis and cell migration in cervical cancer. Based on the docking analysis, the binding ability of chitosan ascorbate nanoparticles with potent cervical cancer protein targets was confirmed. The interactions of molecules concluded that CANs mimic the binding of substrate to these enzymes. Wang et al., 2010 [40], applied Monte Carlo Simulations to design nanoparticles for enhanced tumor targeting. The modeling was applied on three different parameters and it was concluded that, by increasing numbers of ligands, binding energy and length of polymer chain of nanoparticles, specific tumor targeting for longer periods could be achieved. Yang et al., 2010 [298], investigated the physical translocation of nanoparticles with different shapes and volumes across a lipid bilayer by applying computer simulations. In spherical shape, the hydrophobicity and large size of nanoparticles resulted in enhanced permeation and targeting through optimum binding energy. By contrast, in pin like shape, the energy was not required but the size was small. Duncan et al., 2015 [299], applied computer simulation to nanoparticle drug delivery for selective targeting. This study was based on the affinity and selectivity of nanoparticles towards cancerous cells and represented with β. When β is equal to −1, it indicates high selectivity and weak binding to cancer cells. When β = 1, it indicates low selectivity and strong binding, while, when β = 0, it indicates a balance between binding and selectivity which is optimal for cancer cell targeting (Figure 7D). The results demonstrated that nanoparticles offered more selectivity towards cancer cells compared to the normal cell. In addition, nanoparticles showed enhanced binding with cancer cells and slight or negligible binding to normal cells.

Khoshoei et al., 2020, have recently highlighted the applications of molecular dynamic simulation in pH-responsive and stimuli-responsive drug delivery systems for cancer chemotherapy [300]. The approach of molecular dynamic simulation can play a key role in predicting the maximum therapeutic effects through novel drug delivery systems with the least side effects and various other biomedical applications. In addition, the molecular dynamic simulation will provide a smart research-based platform by achieving the aforementioned goals with minimal experimental, human- and animal-based clinical trials. MD simulation studies are very important to underpin the molecular-level understanding of the interaction of anticancer drugs at the target sites, which is very difficult to uncover using experimental approaches.

## 4. Conclusions

A variety of chemotherapeutic agents have been discovered for the management of different types of cancers, but the foremost deficiency associated with chemotherapy is the deprivation of target-specific localization and neoplasia-specific cytotoxicity through chemotherapeutic agent(s). Tremendous advancements in the field of drug delivery systems have been made to achieve safe and effective chemotherapy based on tumor-specific localization, with minimal or no effects on the normal cells but with added effects of improved bioavailability. Stimuli-responsive drug delivery systems are the novel oncotargeted therapeutic moieties for achieving stealth effects, cascaded drug delivery followed by EPR, entry into the cancer cells through overexpression of oncoreceptors and theranostic applications. The aforementioned goals were achieved through the use of temperature, magnetic/electric field, ultrasound, light, pH and enzyme(s), in single or multiple stimuli-responsive drug delivery systems in accordance with the cancer microenvironment; such goals have been critically discussed in this review. Furthermore, the mechanistic approaches associated with fulfilment of objectives by employing cancer biomarkers, improvement in the bioavailability of therapeutic agents, and enhanced apoptosis and necrosis of cancer cells has also been addressed. The review also highlighted the enhanced in vitro and in vivo targeting and internalization of the anticancer agent(s) through the triggered release of drug in cancer-simulated conditions or tumor sites, through steric stabilization of lipid-based systems and by improving drug bioavailability through modifying and/or controlling the drug release from the system. Moreover, the stimuli-responsive drug delivery strategies alleviate drug efflux through lipid-based systems by achieving the biological membrane-like structure and extending the retention time of the anticancer agents at the cancer microenvironment through enhanced permeation and retention (EPR) effect through the use of stimuli-responsive prodrugs, cross linkers, lipids and carriers. All the above mentioned parameters collectively result in the production of reactive oxygen species (ROS), protection against further invasion and metastasis of cancers, reduction in the cancer cell viability, incessant apoptosis of cancer cells and/or tumor site, plummeting of the cancerous mass followed by decreased tumor size and management of different types of malignancies through novel stimuli-responsive drug delivery strategies.

In the future, the conditions including suitable size, surface charge, stealth effects, sterilization and similarity with the biological membrane of the drug delivery systems/nanomedicines required for the achievement of targeting should also be addressed. Moreover, the detailed description regarding advanced in silico modeling and docking studies should also be addressed for the process optimization and achievement of enhanced tumor-specific localization and drug release. In addition, the updated knowledge regarding the characteristics of the cancer microenvironment should also be utilized for addressing (and achieving) safe, effective and economic chemotherapy through advanced multiple stimuli-responsive nanomedicines and drug delivery systems. In short, the current review can provide sufficient shreds of evidence regarding the management of various types of cancers with enhanced cancer-based selectivity and minimal side effects. Moreover, the current review may help to support the work of the drug delivery scientists and the researchers involved in the field of oncogenesis for future decades.

## Figures and Tables

**Table 1:** Stimuli-Responsive Drug Delivery Systems for Cancer Chemotherapy.

S. No	Stimulus	Drug	Drug Delivery System	Reference
1.	Temperature	Doxorubicin	Liposomes, Micellar nanoparticles, Hydrogels, Polymeric nanoparticles, Dendrimers	[25,26,27,28,29]
		Docetaxel	Micelles, Hydrogel, Liquid Suppository, Liposomes	[30,31,32,33]
2.	Magnetic Field	Doxorubicin	Magneto-liposomes, FeCo/Graphite shell Nanocrystals, Alginate embedded Magnetic Nanoheaters, Magnetic iron oxide nanoparticles	[34,35,36,37]
		Docetaxel	Docetaxel grafted magnetic nanoparticles, Nanocomposite, Polymeric iron oxide nanoparticles	[33,38,39]
3.	Electric Field	Antisense oligonucleotides	Liposome nanoparticles, Hydrogels	[40,41]
4.	Ultrasound	Doxorubicin	Polypeptide doxorubicin nanoconjugates, Alginate nanodroplets, PEGylated Liposomes, Microbubbles	[42,43,44,45]
		Docetaxel	Nanobubbles, Lipid microbubbles,	[46,47]
5.	Light	Doxorubicin	Gold nanospheres, Stealth Liposomes, Micelles, Mesoporous silica nanocarriers, Nanogels,	[48,49,50,51,52]
		Docetaxel	PEGylated Gold Nanorod Coated Poly(l-lactide) Microneedles, Nanocomposites,	[53,54]
6.	pH	Doxorubicin	Nanogels, Liposomes, Magnetic chitosan nanoparticles, Microgels, Micelles, Mesoporous silica nanoparticles, Magnetic nanoparticles, Dendrimers	[55,56,57,58,59,60,61,62]
		Docetaxel	Liposomes, Lipid polymer hybrid nanoparticles, Mesoporous carbon nanoparticles, Micelles	[63,64,65,66]
7.	Enzymes	Doxorubicin	Magnetic iron-oxide nanoparticles, Polymer-peptide-drug conjugates, Nanofibers, Dendrimers	[42,67,68,69]
		Paclitaxel	Polymeric nanoparticles, Solid lipid nanoparticles, Dendrimers, Micelles	[70,71,72,73]

**Table 2:** Stimuli-Responsive Lipid-Based Drug Delivery Systems for Cancer Chemotherapy.

S. No	Stimulus	Drug	Lipids	Drug Delivery System	Targeting	Major Findings	Reference
1.	Temperature	Doxorubicin	DPPC:HSPC:Chol:DPPE-PEG	Liposomes	Passive	Hyperthermia assisted rapid drug release and enhanced in vitro cytotoxicity.	[110]
		5-fluorouracil	Lauric acid (LA):oleic acid (OA):linoleic acid (LIA)	SLN	Passive	Mild hyperthermia (39 °C) based quick drug release in diffusion-controlled manner thus showed 2–3 times higher cytotoxicity against cancer cells.	[111]
		Camptothecin	DPPC:DPPG	Lipid-coated nanoparticles	Passive	The formulation showed thermoresponsive controlled drug release, greater cytotoxicity and synergistic activity against cancer cells.	[112]
		Methotrexate	DPPC:DSPC	Liposomes	Passive	Delayed tumor growth and 4–6 fold improved cytotoxicity than free methotrexate through developed Liposomes	[113]
2.	Magnetic field	Docetaxel	DPLC:DOPE:TMAG/FeFe_2_O_4_	Magnetoliposomes	Dual active and passive	These magnetoliposomes showed the dual hyperthermia and magnetic field-assisted enhanced release and cytotoxicity of anticancer agent.	[114]
		Tegafur	DPPC:MPPC:DSPE-PEG_2000_	Liposomes	Passive	The result of these magnetic field sensitive liposomes of tegafur showed greater stability and specificity towards tumor cells.	[115]
		Doxorubicin	DPPC:Chol:DSPE-PEG_2000_-Folate	Lipid-coated nanoparticles	Dual active and passive	Enhanced tumor-specific cytotoxicity, cell uptake and synergistic effects of biological and magnetic field-assisted targeting by magnetic field responsive liposomes relative to non-magnetic liposomes.	[116]
3.	Electric field	Iron oxide nanoparticles	POPC:Chol-γ-Fe_2_O_3_	Nanoparticles	Passive	Efficient and novel method to manufacture SPIONs for effective targeting the tumor cells by applying an external stimuli.	[117]
		Calcein	EPC-P(HEA-HDA-CEA)	Liposomes	Passive	Tumor-specific targeting and triggered drug release from electric field-responsive liposomes for dermal and transdermal drug delivery.	[118]
		Doxorubicin	EPC:CHOL/Fe_3_O_4_	Liposomes	Passive	3–4 fold higher DOX concentration through DOX loaded liposomes at the tumor. Moreover, decrease tumor growth and suppressed lung metastasis through target specific localization of DOX was achieved.	[119]
4.	Ultrasound	Doxorubicin	DPPC, Chol, DSPE-PEG_2000_-amine, α-tocopherol, & (PFC_5_)	Nanoliposomes	Passive	Perfluoropentane and DPPC based DOX loaded liposomes showed controlled and target-specific release upon insonation with low-intensity ultrasound.	[120]
		Vincristine	HSPC:DSPE-PEG_2000_/DSPE-PEG2000-Mal/HMME	Emulsion liposomes (eLiposomes)	Passive	Site-specific delivery of vincristine triggered by ultrasound. In addition, the ultrasound also caused ROS-based tumor cell death through triggered release of drugs.	[121]
		Doxorubicin	DOPE:DSPC:DSPE-PEG:Chol	Liposomes	Passive	These ultrasound-sensitive liposomes showed long blood circulation of drug for higher tumor uptake.	[122]
5.	Light	Doxorubicin	DPPC:HSPC:Chol:DSPE-PEG2000/OMP-HauNS	Liposomes	Dual active and passive	These liposomes showed a promising delivery of chemotherapeutics as light-triggered targeted drug release.	[123]
			DPPC:DC_8_,_9_PC:DSPE-PEG_2000_	Lipid nanoparticles	Passive	Spatial and temporal release of therapeutic agents at tumor site and preferable taken up by permeable tumor vasculature was achieved and tumor-specific targeting was achieved by developed liposomes.	[124]
			ICG-ODA,S100, PLsPC,Chol,DSPE-PEG_2000_ & DSPE-PEG_2000_-NH-DSC	Nanoparticles	Dual active and passive	These light-responsive nanoparticles structured with s light-responsive lipids showed tumor-specific DOX release and tumor growth inhibition compared to chemotherapy alone.	[125]
			DPPC,HSPC,Chol,DSPE-PEG_2000_-NH-maleimide, OMP-HAuNS, HER2ab	Liposomes	Dual active and passive	Controlled and photo thermal release of DOX through NIR-responsive liposomes showed greater cytotoxicity than DOX nanoparticles alone. Moreover, DOX in conjunction with NIR laser showed significant antitumor efficacy.	[126]
6.	pH	Cytosine-h-D-arabinofuranoside	PC:CHEMS:T-80:OAlc Or DOPE:CHEMS	Liposomes	Dual active and passive	These liposomes showed excellent stability at pH 7.4 and rapid destabilization upon acidic environment of cancer cells thus showed greater and targeted drug release.	[127]
		DNA plasmid	DOPE:C-DOPE/FA-PEG-DOPG	Liposomes	Dual active and passive	pH-dependent release of endosome entrapped DNA into the cytoplasm and tumor-specific targeting was achieved with limited cytotoxic effects on normal cells.	[128]
		Curcumin & Paclitaxel	DOPC:DOPE:Cholesterol: DSPE-PEG_2000_	Lipid-coated nanoparticles	Passive	The acidic pH-based tumor-specific targeting and tumor inhibition was achieved.	[129]
		Docetaxel	PE/CHOL/CHEMS/RGD-CHEMS	Liposomes	Dual active and passive	Enhanced tumor-specific delivery compared to conventional pH-sensitive liposomes was achieved. Moreover, the higher uptake of drug by cancer cells was also achieved.	[130]
7.	Enzyme	siRNA	DSPE-PEG_2000_/DSPE-PEG_2000_ angiopep/DOTAP/POPC/DODAP/DOPE-Rhb	Lipid nanoparticles	Dual active and passive	These lipid nanoparticles of siRNA showed safe, stable and effective delivery for treating the central nervous system disorders.	[131]
		Oxaliplatin	DPPC:DPPG:DSPC:DSPG: DSPE-PEG_2000_:HSPC:Chol	Nanoparticles	Passive	These liposomes showed improved anti-tumor efficiency towards the enzymes directed by lysolipids and serum protein.	[132]
		Insulin	PC:DPPE:Chol:OA/PA/SA	Liposomes	Passive	These enzyme-triggered pH-sensitive liposomes showed improved tumor specificity, rapid release at cancer cells and ability of a drug to reach the systemic circulation in a controlled manner.	[133]

**Table 3:** Biocompatible Thermoresponsive Lipids and their Phase Transition Temperature.

S. No	Lipids	Abbreviation	Transition Temperature Tc (°C)	References
1.	Dipalmitoyl phosphatidylcholine	DPPC	41 °C	[135]
2.	Dipalmitoyl phosphatidylglycerol	DPPG	41 °C	[135]
3.	Dimyristoyl phosphatidylserine	DMPS	38 °C	[139]
4.	Egg spingomyline	ESM	40 °C	[140]
5.	Dipalmitoyl phosphatidylserine	DPPS	51 °C	[141]
6.	Hydrogenated soybean phosphatidylcholine	HSPC	52 °C	[142]
7.	Dimyristoyl phosphatidylethanolamine	DMPE	50 °C	[143]
8.	Dimyristoyl phosphatidylcholine	DSPC	55 °C	[144]
9.	Dimyristoyl phosphatidylglycerol	DSPG	55 °C	[145]
10.	Dipalmitoyl phosphatidylethanolamine	DPPE	60 °C	[146]

**Table 4:** List of FDA-Approved Prodrugs and Liposomes for Anticancer Effects.

Product Name	Drug Delivery System	Drug	Indication	Clinical Status	Reference
Istodax	Prodrug	Romidepsin	Cutaneous T-cell Lymphoma	FDA (US) approved in 2009	[166]
Zytiga	Prodrug	Abiraterone acetate	Metastatic castration-resistant prostate cancer	FDA (US) approved in 2011	[167]
Rituxan	Prodrug	Rituximab	β-cell non-Hodgkin’s lymphoma and Refractory low-grade lymphoma	FDA approved	[168]
Mylotag	Prodrug	Gemtuzumab ozogamicin	Acute myeloid leukemia	US and EU approved in 2018	[169]
Erbitux	Prodrug	Cetuximab	Colorectal cancer	FDA approved in 2009	[170]
Doxil	Liposomes	Doxorubicin	Ovarian and breast cancer, Kaposi’s sarcoma	FDA approved in 1995, EMA approved in 1996	[171]
Myocet	Liposomes	Doxorubicin	Metastatic breast cancer	FDA approved in 2000	[172,173]
Lipodox	Liposomes	Doxorubicin	Kaposi’s sarcoma, breast and ovarian cancer	FDA approved in 2013	[174,175]
Lipusu	Liposomes	Paclitaxel	Solid tumor and ovarian cancer	FDA approved in 2006	[176,177]
DaunoXome	Liposomes	Daunorubicin	Hematological malignancy and Kaposi’s sarcoma	FDA approved in 1996	[178,179]
Onivyde	Liposomes	Irinotecan	Metastatic pancreatic cancer and multiple solid tumor	FDA approved in 2015	[180,181]
Marqibo	Liposomes	Vincristine sulfate	Acute lymphoblastic leukemia	FDA approved in 2012	[182,183]
DepoCyt	Liposomes	Cytarabine	Neoplastic meningitis, lymphoma and solid tumor	FDA approved in 2007	[184,185]
Eligard	Liposomes	Leuprolide acetate	Prostate cancer	FDA approved in 2002	[186,187]
Mepact	Liposomes	Mifamurtide	Non metastatic osteosarcoma,	EMA approved in 2009	[188,189]

**Table 5:** pH Associated with Different Cellular Compartments and Tissues (Adopted from [192,193,199]).

Cellular Compartment/Tissue	pH
Early endosome	6.0–6.5
Late endosome	5.0–6.0
Lysosome	4.5–5.0
Golgi	6.4
Stomach	1.0–3.0
Duodenum	4.8–8.2
Colon	7.0–7.5
Blood	7.35–7.45
Tumor	6.5–7.2

**Table 6:** Stimuli-Responsive Prodrugs for Cancer Chemotherapy.

S. No	Stimulus	Drug	Prodrug	Targeting	Major Findings	Reference
1.	Temperature	Doxorubicin	mPEG_5000_-b-PMAmLac_2_-r-AzEMA)-DOX-propGA_3_	Dual active and passive	Improved in vitro and in vivo cytotoxicity of cancer cells was achieved through hyperthermia in the presence of beta glucuronidase.	[206]
			P-cypate/P-DOX	Passive	The micelles showed enhanced in vitro drug release at acidic pH. Micelles also displayed both in vitro and in vivo cytotoxicity at cancer cells in the presence of light source and the light source caused hyperthermia. So collectively micelles showed triple stimuli responsiveness.	[52]
			P(MAOEPC)-b-P(MEMA)-Hz-DOX + IR-780 (PDOX/IR-780)	Passive	Enhanced pH and photothermal in vitro drug release, cytotoxicity and drug internalization at tumor site and cancer cells was achieved by using pH-responsive polymeric prodrug micelles and NIR light source.	[207]
			PNHNH_2_-b-POEGMA-FA-DOX + IR-825 (PDOX/IR-825)	Dual active and Passive	Enhanced in vitro and in vivo cytotoxicity of cancer cells was achieved at acidic pH in the presence of light source.	[208]
		Cisplatin	P(Pt-Cyp-MEO_2_MA-co-MASI)-b-PHPMA	Passive	Enhanced photothermal responsiveness, drug efflux and in vitro cytotoxicity was achieved.	[209]
		Camptothecin	Nap-CPT-Ad + HA-CD + IR-825	Passive	Enhanced in vitro and in vivo cytotoxicity of cancer cells was achieved through light and temperature chemotherapeutics.	[210]
2.	Magnetic Field	Doxorubicin	DOX-Gd(III)	Passive	Enhanced magnetic field and pH-responsive in vitro drug release was achieved.	[211]
		Cisplatin	poly(I:C)-Pt(IV)-IONPs	Passive	Enhanced in vitro cytotoxicity using various cancer cell lines and immune cell-facilitated anti-neoplastic effects were achieved.	[212]
			HSPt–PEG-SPIONs	Passive	Enhanced in vitro and in vivo cytotoxicity of cancer cells and theranostic application of Pt(IV) prodrug-loaded superparamagnetic iron nanoparticles (nanocomposites) was established.	[213]
3.	Electric field	5-aminolaevulinic acid (ALA)	ALA + Iontophoresis	Passive (diffusion)	Enhanced efflux of ALA across the skin and in dermis through iontophoresis was achieved.	[214]
4.	Ultrasound	Doxorubicin	Heparin-FA-PEG-cRGD-DOX-MB	Dual active and Passive	Enhanced in vitro and in vivo cytotoxicity of cancer cells through combination of ultrasound and pH responsiveness was established.	[215]
			per-fluoro-pentane/C_9_F_17_-Pasp(DET)/cis-aconityl-DOX/PGA-g-mPEG_n_-prodrug nanodroplets	Dual active and Passive	Enhanced in vitro cytotoxicity of cancer cells and theranostic application was achieved.	[216]
5.	Light	5-aminolaevulinic acid (ALA),	Ac-LPheALAOMe	Dual active and Passive	Enhanced stability, hydrophilicity photosensitization and cancer cells uptake were achieved.	[217]
		Coumarin	D-L-dps/hυ	Passive	Enhanced photosensitization, quick in vitro drug release and singlet oxygen-cleavable onco-cytotoxicity in the presence of specific light intensity was achieved.	[218]
		Gemcitabine	GEM-L-mTPP/658 nm Light source	Passive Targeting	Enhanced photosensitization, singlet oxygen-cleavable cross liker based in vitro cytotoxicity, cascaded drug release at tumor site and combination of photodynamic therapy and chemotherapy was achieved in the presence of suitable light source.	[219]
		Paclitaxel	FA-PEG_2000,5000_-Pc-L-PTX	Dual active and Passive Targeting	Achievement of amphiphilic prodrug with enhanced light-mediated oncoreceptors overexpression, improved cytotoxicity associated with suitable chain length of ligands and enhanced cellular uptake was established in the presence of photosensitizer and singlet oxygen-cleavable cross linkers.	[220]
		Cabazitaxel	Ppa⸞CTX-S/Se-OA⸞DSPE-PEG_2000_ NPs	Passive	Enhanced in vitro cytotoxicity, cellular uptake and intracellular release of anticancer agent was achieved in the presence of light source (of suitable wavelength).	[221]
6.	pH	Doxorubicin	PMAC-graft-(ADPC-co-Mal-DOX	Passive	Enhanced drug internalization, cellular uptake and in vitro cytotoxicity at cancer pH was observed in the presence of esterases (dual pH and enzyme responsiveness).	[222]
			ADPC-PEI-Mal-DOX	Passive	Improved in vitro drug release, cytotoxicity, internalization and cellular uptake was achieved at acidic pH.	[223]
			mPEG-b-PLA-g-DOX	Passive	Enhanced in vitro release and cytotoxicity of doxorubicin at acidic pH and cancer cells were established.	[224]
			FA-BSA-CA-DOX	Dual active and Passive	Enhanced tumor selectivity, efficacy, in vitro cytotoxicity and release of doxorubicin at the tumor site and cancer pH was achieved.	[225]
		Curcumin	Cu-Dex micelles	Passive	Quick in vitro release and enhanced cytotoxicity at tumor site and cancer pH was established.	[226]
7.	Enzyme (s)	Chemotherapeutic agent (s)	Antibody directed enzyme prodrug therapy, Gene directed enzyme prodrug therapy and Glutathione transferases based prodrug therapy	Dual active and Passive	N/A *	[227,228]

* N/A represents not available or not applicable.

**Table 7:** Stimuli-Responsive Carrier/Polymer-based Drug Delivery Systems for Cancer Chemotherapy.

S. No.	Stimulus	Drug	Polymer	Drug Delivery System	Targeting	Major Findings	Reference
1.	Temperature	Doxorubicin	Poly (*N*-isopropylacrylamide-*co*-*N*, *N*-dimethylacrylamide)-*b*-poly (D, L-Lactide-*co*-glycolide)	Micelles	Passive	Thermosensitive doxorubicin-loaded micelles showed greater cytotoxicity at the temperature above the lower critical solution temperature.	[252]
			Poly (*N*-isopropylacrylamide)	Hydrogel	Passive	Thermoresponsive Poly (*N*-isopropylacrylamide) hydrogels have good optical transparencies, mechanical properties and better swelling and deswelling properties.	[253]
		5-fluorouracil	Poly (*N*-vinyl caprolactam) based chitosan	Polymeric nanoparticles	Passive	Thermosensitive Poly (*N*-vinyl caprolactam) based chitosan 5-FU nanoparticles showed increased apoptosis to tumor cells.	[254]
2.	Magnetic field	Biotin and Doxorubicin	Poly (ethylene glycol)	Magnetic nanoparticles	Dual active and Passive	Super paramagnetic norbornene copolymers biotin and doxorubicin are potential theranostic agents for tumor diagnosis.	[255]
		Oxanorbornene	(Poly 1-Poly 7,With C16 homo block and Co-*r*-Fe)	Magnetic nanoparticles	Passive	The dipolar interactions of the cobalt nanoparticles with the phase-separated domains were responsible for the phenomenon of room temperature ferromagnetic particles of the block co-polymers.	[256]
3.	Electric Field	N/A *	Poly (vinyl alcohol) (PVA)/Poly(acrylic acid) (PAA)	Hydrogels	Passive	Poly (vinyl alcohol) (PVA)/Poly(acrylic acid) (PAA) IPN hydrogels showed bending behavior upon exposure of electric field.	[257]
			Bacterial Cellulose	Actuator	Passive	Bacterial cellulose actuator as an electro-active bio-polymer for implantable devices in wet environments.	[258]
			chitosan and poly (hydroxyethyl methacrylate)	Hydrogels	Passive	Upon the exposure of electric field, the degree of bending deformation increases.	[259]
4.	Ultrasound	N/A *	Gelatin	laser therapy	Passive	Fibrinolysis promoted by injecting fibrinolytics deeply into thrombus by means of Ho:YAG laser-induced liquid jet.	[260]
		Paclitaxel	poly (ethylene oxide)-co-poly(L-lactide) (PEG-PLLA) or poly (ethylene oxide-co-polycaprolactone (PEG-PCL)	Nanoemulsion/Microbubbles	Active	Upon exposure of ultrasound, the drug-loaded nanoemulsion is converted into microbubbles (in-situ).	[261]
		Doxorubicin	Poly (ethylene oxide)-co-poly(prolylene oxide)-co-poly(ethylene oxide), poly(prolylene oxide) (PPO)	Micelles	Passive	Enhanced cell viability, sensitization of multi-drug resistant cells, efflux, and cancer cell killing in presence of ultrasound stimuli due to pluronic triblock copolymer.	[262]
5.	Light	N/A *	Cinnamic acid as cinnamon derivative		Passive	Enables the photo-induced shape changes in response to light.	[263]
			Cinnamylidene acetic acid as cinnamon derivative		Passive	The exposure of light on the cinnamon derivative results in higher yield of cross-linked polymers and less swelling behavior.	[264,265]
		Fibrin	PEG_7500_	Gold nanoparticles	Dual Active and Passive	Gold nanoparticles have significant effect on disease state in deep vein thrombosis via local and catheter-delivered approaches.	[266]
6.	pH	5-fluorouracil	Poly (*N*-isopropylacrylamide)/carboxymethyl chitosan	Injectable hydrogels	Passive	In situ cross-linked depot injectable hydrogels are dual pH and thermoresponsive having the potential of onco-intracellular drug delivery through parenteral delivery.	[267]
		Heparin	Chitosan	Polymeric nanoparticles	Passive	PH-sensitive chitosan/heparin nanoparticles can infiltrate the cell-to-cell junction and also adhere locally with the H-pylori at the infection site in the stomach.	[268]
			Poly (*N,N*-diethylacrylamide-co-acrylic acid)	Hydrogel	Passive	Thermo and pH dual-responsive grafted hydrogels showed better thermo-reversible behavior as compared to normal hydrogels.	[269]
7.	Enzyme	N/A *	Dextran	Polymeric nanoparticles	Active	Dextrin-phospholipase A_2_ showed reduced toxicity and α-amylase triggered activity.	[270]
		Doxorubicin	*N*-(2-hydroxypropyl) methacrylamide (HPMA)	Polymeric nanoparticles	Passive	Polymer conjugates have combine effect of endocrine therapy and chemotherapy.	[271]
		N/A *	Perthiolated β-cyclodextrin	Gold nanoparticles	Active	Sensitivity of nanoparticles biosensors enhanced by assembling through supramolecular multi-valent interactions.	[272,273]

* N/A represents not available or not applicable.

## Data Availability

The data presented in this study are openly available.
